# Solution-Tunable Interfacial Interaction Landscape Governs Anomalous Nanoparticle Diffusion in Liquid-Phase Electron Microscopy

**DOI:** 10.1021/acsnano.6c04149

**Published:** 2026-05-01

**Authors:** Isabel Panicker, Zain Shabeeb, Cory Hargus, Vida Jamali

**Affiliations:** † School of Chemical and Biomolecular Engineering, 1372 Georgia Institute of Technology, Atlanta, Georgia 30332, United States of America; ‡ 831866 Entalpic, 5 Parv. Alan Turing, Paris 75013, France

**Keywords:** anomalous diffusion, single-particle tracking, passive nanorheology, liquid-phase transmission electron microscopy, interfacial transport

## Abstract

Understanding how nanoparticles move near liquid–solid interfaces is central to nanoscale transport in catalysis, biology, and soft materials. Here, we uncover the physical mechanisms governing anomalous surface diffusion of PEG-coated gold nanorods (AuNRs) near the silicon nitride (SiN_*x*_) membrane in liquid-phase transmission electron microscopy (LPTEM). By systematically tuning the ionic environment (H_2_O, 5 mM H_2_SO_4_, 1.5 mM NaCl, 5 mM PBS), we show how electrostatic screening and ion-specific surface interactions modulate the interaction landscape, altering the strength and abundance of binding sites that govern the confinement and mobility of nanoparticles. Statistical analyses and deep learning classification of particle trajectories reveal a tunable transition between fractional Brownian motion (FBM) in strongly interacting systems (H_2_O, H_2_SO_4_) and annealed transient time motion (ATTM) in screened environments (NaCl, PBS). These results establish electrostatic screening and specific ion effects as external controls that program near-surface transport, shifting the diffusion mechanism from FBM to ATTM and tuning the particle mobility. To further elucidate the interfacial dynamics, we introduce a passive nanorheology framework in LPTEM, modeling the near-surface environment of FBM-classified conditions as an effective viscoelastic medium. Leveraging translational and rotational trajectories as nanoscale rheological probes, we reconstruct frequency-dependent viscoelastic moduli to extract relaxation times and elastic-to-viscous crossover moduli that report on interaction strength at the SiN_*x*_ interface. Together, these advances provide both control and diagnosis of interfacial mechanical response in LPTEM, positioning it as a quantitative tool for probing nanoscale transport in complex soft-matter and interfacial systems.

## Graphical abstract

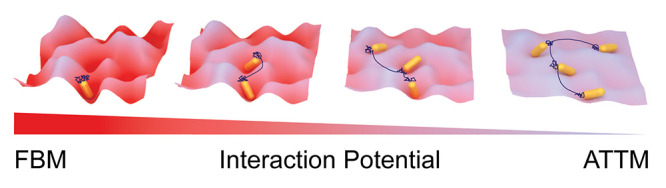

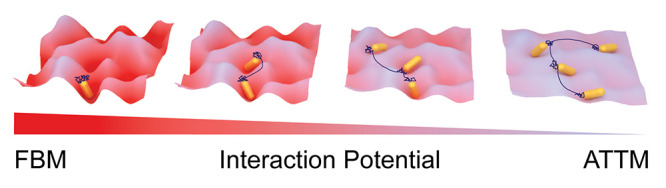

Single-particle tracking (SPT) probes the mechanical properties of complex media by analyzing the trajectories of individual particles diffusing in the medium. The resulting trajectories can reveal heterogeneities in motion, uncovering multiple coexisting dynamical states within the same system.^1^ Observing transitions between free diffusion, confinement, subdiffusion, or directed transport enables linking particle motion to underlying molecular interactions, such as transient binding or confinement in viscoelastic microenvironments.^2^ These capabilities make SPT an indispensable tool for interrogating nanoscale environments where thermal fluctuations, crowding, and surface interactions strongly govern dynamics. SPT has illuminated processes ranging from protein–DNA interactions in cells to polymer viscoelasticity to catalytic nanoparticle transport, demonstrating its power to connect tracer motion to system function.^1,2^ However, traditional SPT is typically performed using optical microscopy, whose diffraction-limited spatial resolution precludes capturing the sub-100 nm dynamics governed by rapid thermal fluctuations and nanoscale surface-dominated interactions.

Liquid-Phase Transmission Electron Microscopy (LPTEM) overcomes the spatial resolution limit of optical microscopy, enabling SPT with nanometer spatial resolution.^3–9^ This capability allows for direct visualization of nanoparticles diffusing at liquid–solid interfaces, providing access to nanoscale spatial dynamics under realistic solution conditions. However, numerous studies have reported that nanoparticles exhibit anomalous (i.e., non-Brownian) subdiffusive motion in aqueous solutions near the silicon nitride (SiN_*x*_) membrane of the liquid cell and other charged liquid-cell membranes, with diffusion coefficients often orders of magnitude slower than expected with Brownian motion in bulk aqueous environments.^10–32^ This anomalous subdiffusion is attributed to a complex interplay of surface-mediated interactions, including electrostatic and van der Waals forces, that arise between the particle and the surface.^11,14–21,23–27,29,31–36^ Collectively, these forces give rise to a heterogeneous interfacial energy landscape characterized by localized potential wells whose depths may exceed thermal energy, leading to near-surface diffusion that deviates from classical Brownian motion.^32^

To establish LPTEM as a robust SPT-based method, it is crucial to understand the origins of this anomalous diffusion and the underlying physical mechanisms at play, as well as to develop independent external controls that can both elucidate these mechanisms and enable deliberate modulation of particle dynamics within the liquid cell. Herein, we investigate how tuning the ionic environment of the aqueous medium in the liquid cell of LPTEM modulates the SiN_*x*_ membrane’s surface chemistry and consequently influences nanoparticle motion. We use neutrally charged 75 nm × 25 nm (length × diameter) polyethylene glycol (PEG)-coated gold nanorods (AuNRs) as a model system and study their surface diffusion in four conditions: pure water, 5 mM H_2_SO_4_, 1.5 mM NaCl, and 5 mM phosphate buffered saline (PBS). We selected PEG-coated AuNRs as charge-minimized, sterically stabilized particles. 5 mM H_2_SO_4_ is selected as a strong-acid condition to preset a proton-rich, low-pH interfacial environment that is directly motivated by beam-induced acidification in LPTEM and is expected to shift silanol acid–base chemistry, thereby providing a targeted test of proton-mediated changes in the diffusion of nanoparticles. 1.5 mM NaCl and 5 mM PBS are selected to preset, respectively, a monovalent screening condition with minimal specific-ion chemistry and a multivalent condition that can additionally promote specific adsorption/passivation on SiN_*x*_. These conditions are chosen to systematically modulate the pH, ionic strength, and electrostatic screening at the liquid–solid interface, allowing us to probe how surface charge and ion-mediated interactions influence nanoparticle mobility and the nature of anomalous diffusion. In all ionic solutions tested, we observe enhanced mobility of AuNRs near the SiN_*x*_ membrane of the liquid cell relative to pure water. The nature of their diffusive behavior and underlying interaction dynamics, however, notably differ depending on whether the solution modulates the surface charges through pH effects, electrostatic screening, or specific ion adsorption.

To classify the underlying mechanisms of anomalous diffusion, we use a deep learning-based diffusion classifier, MoNet 2.0,^32,37^ which reveals that the surface diffusion mechanism changes depending on the interaction landscape of the liquid–solid interface. Specifically, we observe a transition from viscoelasticity-driven fractional Brownian motion (FBM)^38,39^ to time-dependent-diffusivity-driven annealed transient time motion (ATTM) which is a class^40,41^ of weakly nonergodic subdiffusion that has not been previously reported in LPTEM studies. We further analyze these dynamics by conducting passive nanorheology on nanoparticle trajectories to quantify the effective viscoelastic response experienced by the nanoparticles at the liquid–solid interface. By modeling the resulting FBM-classified particle trajectories as motion within an effective single-relaxation-time Maxwell fluid, where nanoparticle-surface interactions provide an elastic restoring force and the confined liquid layer contributes viscous dissipation, we interpret the observed dynamics as an emergent viscoelastic response characterized by a single relaxation time. These results show that LPTEM-based SPT can capture the mechanical properties of the liquid cell environment at nanometer-length scales, which were previously inaccessible.

By systematically modulating the surface interactions at the liquid–solid interface and classifying the resulting anomalous diffusion modes using deep learning, we uncover how interfacial chemistry dictates distinct modes of anomalous diffusion behavior. This work not only reports the first observation of ATTM in LPTEM but also introduces nanorheology in LPTEM as a quantitative framework that interprets nanoparticle surface diffusion at the SiN_*x*_ interface in terms of an effective interfacial viscoelastic response, providing a generalizable standard for future studies of nanoscale transport. Together, these results establish a foundation for developing LPTEM as a quantitative SPT method for studying nanoscale transport and mechanics in biological, catalytic, and soft polymeric systems.^1,2^

## Results and Discussion

### Medium-Dependent Anomalous Diffusion

Figure 1 shows representative experimental LPTEM acquisitions of PEG-coated AuNRs (PEG *M*_w_ = 1.63 kDa, measured by liquid chromatography–mass spectrometry (LC-MS)) diffusing near the SiN_*x*_ membrane in four diffusion media: pure water (pH = 7.30), 5 mM H_2_SO_4_ (pH = 2.60), 1.5 mM NaCl (pH = 7.03), and 5 mM PBS (pH = 7.73). Corresponding Movies S1–S4 are provided in the Supporting Information. All data were acquired at a magnification of 19.5kx, 1024 × 1024 pixels, 100 frames per second (fps) (binned to 10 fps postacquisition), with an electron flux of 30 e^–^/(Å^2^·s), and an approximate beam radius of 2.5 μm. To avoid irradiation overlap, fields of view were recorded in nonoverlapping regions of the liquid cell under identical flux, standardizing irradiation history across data sets. Videos were acquired promptly upon initial visualization of particles within the field of view. Additionally, to ensure robustness, each medium was examined in two independent experimental replicates acquired under the same flux, magnification, and beam exposure. Trajectories were selected from spatially isolated AuNRs with no detectable particle–particle contacts in the field of view, ensuring the reported dynamics reflect single-particle interfacial coupling rather than collective assembly.

**Figure 1:**
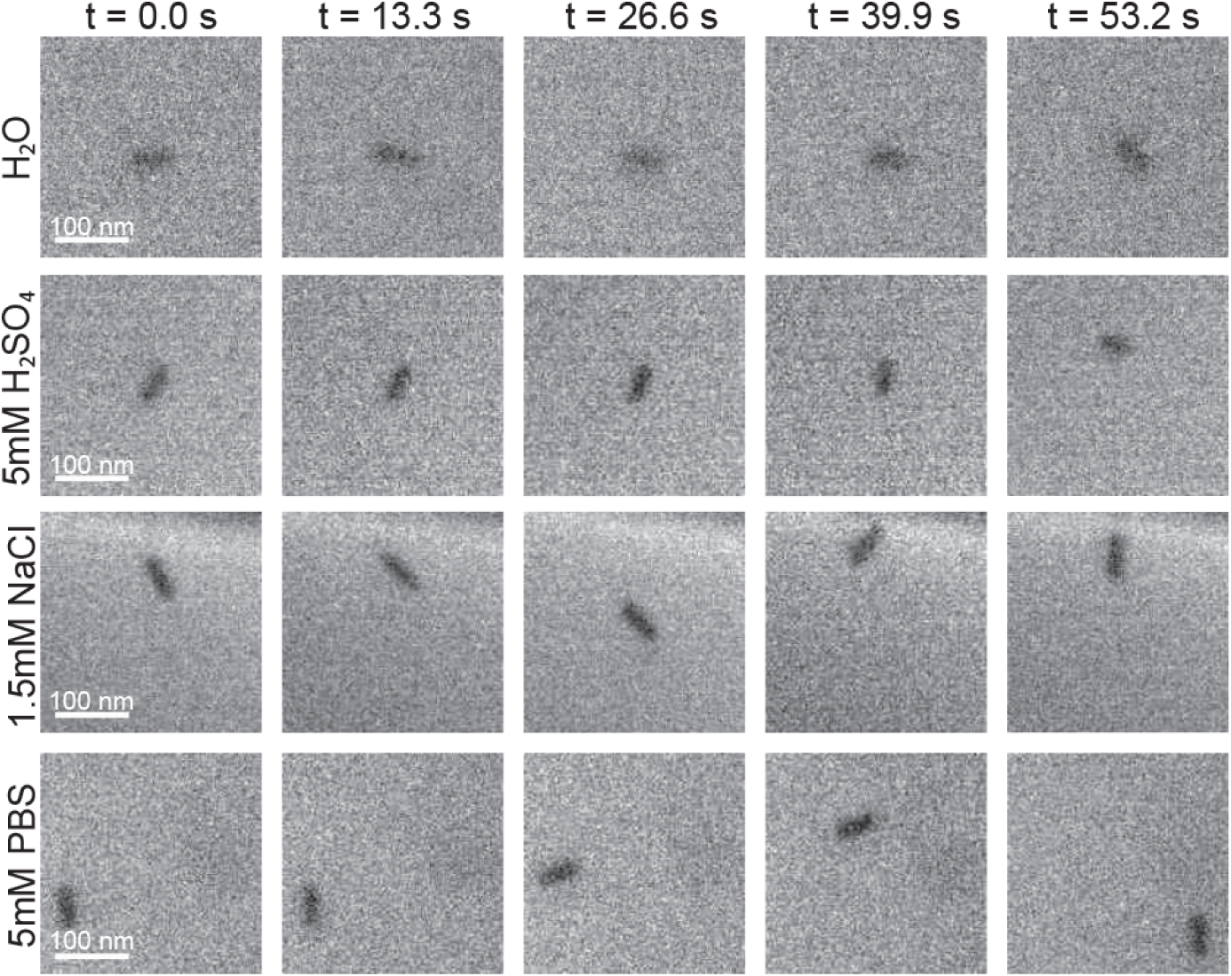
LPTEM image acquisitions of 75 nm PEG-coated gold nanorods diffusing in H_2_O, 5 mM H_2_SO_4_, 1.5 mM NaCl, and 5 mM PBS over time. All acquisitions were performed at 200 kV, 100 fps (binned to 10 fps postacquisition), and acquired at an electron flux of 30 e^–^/(Å^2^·s). Images shown above and in Movies S1–S4 are cropped from 1024 × 1024 pixels (original acquisition image size) to 150 × 150 pixels. The scale bar is 100 nm and is applied to all conditions.

In pure water, AuNRs exhibited minimal translational and rotational motion, indicating strong surface confinement and restricted mobility. In 5 mM H_2_SO_4_ particles displayed slightly higher translational and rotational mobility than in pure H_2_O; however, they still remained bound to the surface. In contrast, AuNRs diffusing within 1.5 mM NaCl and 5 mM PBS exhibited significantly enhanced mobility compared to H_2_O and 5 mM H_2_SO_4_. As AuNRs traversed the membrane surface, they underwent brief, localized slowdowns at interaction sites, followed by continued motion. Among all four media tested, AuNRs in 5 mM PBS displayed the highest apparent mobility.

To quantify these observations, each video was analyzed, and particles were segmented and tracked using SAM-EM, a deep learning-based segmentation and tracking framework for LPTEM videos.^42^ Representative 2D trajectories from each respective solution are shown in Figure 2a. Trajectories of AuNRs diffusing in pure H_2_O span the smallest area, consistent with the lowest diffusivity. In 5 mM H_2_SO_4_, nanoparticle trajectories exhibit confined motion with occasional hops. In 1.5 mM NaCl and 5 mM PBS, the nanoparticle trajectories span much larger spatial areas, with the 5 mM PBS case having the largest diffusion area, indicating the highest observed diffusivity. In both cases of 1.5 mM NaCl and 5 mM PBS, the particles experience frequent transitions between mobile and immobile states.

**Figure 2:**
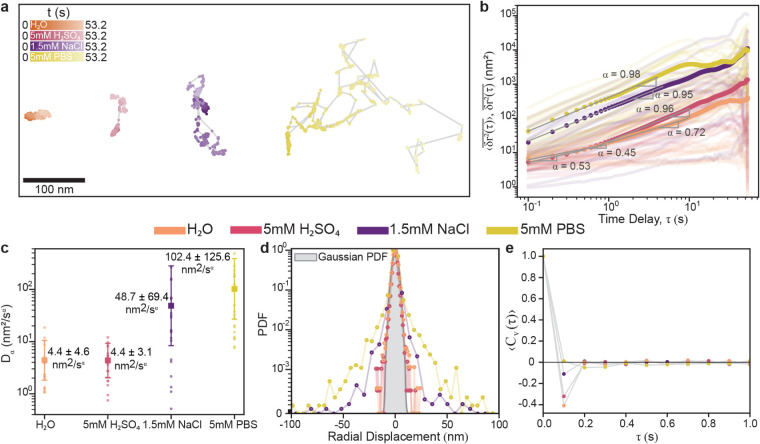
Statistical analysis of AuNR translational diffusion in each respective solution. Color indicates diffusion medium: H_2_O (Orange), 5 mM H_2_SO_4_ (Pink), 1.5 mM NaCl (Purple), and 5 mM PBS (Yellow). (a) Representative 2D trajectories of AuNRs in each medium. Color intensity encodes time [0–53.2 s]. (b) Log–log plot of time-averaged mean squared displacements (tMSDs) shown as transparent solid lines, grouped by medium, with the ensemble-average of tMSDs (e-tMSDs) overlaid as circular markers. Extracted anomalous exponents, α, and power-law fits (gray solid lines) for the short-time and long-time delay regimes are shown separately when applicable. (c) Generalized diffusion coefficients (*D*_α_) obtained from the *y*-intercepts of the e-tMSDs power-law fits overlaid with single-trajectory *D*_α_ values. Whiskers denote one standard deviation from the e-tMSD values. (d) Probability density functions (PDFs) of particle displacements, aggregated for all particles over each time frame (every 0.1 s) of the acquisition, colored by solution media. The gray overlay represents a Gaussian PDF. (e) Ensemble-averaged velocity autocorrelation functions (VACFs) of particles colored by medium.

To characterize the nature of anomalous diffusion in each medium, we computed the time-averaged mean squared displacement (tMSD) for the radial displacements, $\delta r=\sqrt{\delta x^{2}+\delta y^{2}}$ . Here *r* denotes the radial coordinate computed from the particle’s Cartesian positions *x* and *y*. Radial displacement (δ*r*) is defined as the change in the radial position of a particle between consecutive frames. Twenty individual 2D trajectories per condition are obtained evenly from two independent replicates (10 trajectories per replicate), and are all truncated to 532 frames (shortest trajectory size) for consistency (transparent solid lines in Figure 2b). These tMSDs were then averaged over the ensemble of 20 trajectories to arrive at the ensemble-averaged tMSDs (e-tMSD) given by

$$\left\langle \overset{―}{\delta r^{2}\left(\tau \right)}\right\rangle =\left\langle \overset{¯}{{\left(r_{t+\tau }-r_{t}\right)}^{2}}\right\rangle$$ 1

Here, *t* denotes the measurement time and τ denotes the time delay. The (·̅) denotes the time average over the time delay windows of size τ while the ⟨(·)⟩ denotes the ensemble average. (See Supporting Information for a discussion of using eMSD versus e-tMSD for this data set.) The e-tMSD curves were fit to a power-law function, from which the anomalous exponent (α) was extracted as the slope of the corresponding log–log plot. The power-law function fit is given by^43^

2 $$\left\langle \overset{―}{\delta r^{2}\left(\tau \right)}\right\rangle \approx 2dD_{\alpha }\tau^{\alpha }$$

where *d* is the dimensionality of the system, which is 2 in the case of 2D trajectories, and *D*_α_ is the generalized diffusion coefficient obtained from e-tMSD. The e-tMSD curves for AuNRs diffusing in pure H_2_O and 5 mM H_2_SO_4_ do not follow a single power-law scaling across the full time range and instead exhibit two distinct slope regimes with a characteristic crossover time. To account for this, we segmented the curves into short-time and long-time delay regimes and fitted each regime independently to capture differences between short- and long-time diffusion. AuNRs diffusing in 1.5 mM NaCl and 5 mM PBS exhibit e-tMSD curves that are approximately linear on log–log axes, suggesting a single power-law scaling across the entire observation time window. The extracted anomalous exponents, α, are shown in Figure 2b, confirming this trend.

To quantify the statistical robustness of the ensemble scaling behavior, α values were obtained from power-law fits to the ensemble-averaged e-tMSDs, with 95% confidence intervals (CIs) derived from 2000 bootstrap resamplings with replacement (see Table S1).^44^ For AuNRs diffusing in 1.5 mM NaCl and 5 mM PBS, α values are near unity (0.95, 95% CI: 0.83–1.02; and 0.98, 95% CI: 0.93–1.03, respectively). AuNRs diffusing in pure H_2_O exhibit subdiffusive behavior (i.e., α < 1) with an α value of 0.45 (95% CI: 0.29–0.56), at short time delays (<0.9 s) and 0.72 (95% CI: 0.59–0.79), at long time delays. AuNRs diffusing in 5 mM H_2_SO_4_ also show a similar evolution, having an α value of 0.53 (95% CI: 0.44–0.63) at short time delays (<0.5 s) and 0.96 (95% CI: 0.77–1.11) at long time delays.

Figure 2c presents the generalized diffusion coefficients *D*_α_, obtained from power-law fits to e-tMSDs, where the intercept is the *D*_α_. To quantify the statistical robustness of the scaling behavior, the corresponding *D*_α_ values were obtained from the same e-tMSDs and reported as the mean ± one standard deviation of the diffusion coefficients derived from individual tMSDs, capturing the spread among trajectories and the resulting heterogeneity in local diffusivity at the interface. The overall diffusivity of AuNRs increases systematically with the ionic environment, remaining low in pure H_2_O (4.4 ± 4.6 nm^2^/s^α^) and 5 mM H_2_SO_4_ (4.4 ± 3.1 nm^2^/s^α^), and rising markedly in 1.5 mM NaCl (48.7 ± 69.4 nm^2^/s^α^) and 5 mM PBS (102.4 ± 125.6 nm^2^/s^α^). Although the generalized diffusion coefficients in H_2_O and 5 mM H_2_SO_4_ are comparable, the larger anomalous exponent in 5 mM H_2_SO_4_ indicates faster growth of the e-tMSD with time delay, consistent with slightly enhanced mobility within an otherwise confined, subdiffusive interfacial state.

We further analyzed the statistical properties of the trajectories by calculating the probability density function (PDF) of the displacements (Figure 2d) and the velocity autocorrelation function (VACF) (Figure 2e). The PDF of displacements captures the frequency of different radial step sizes over 0.1 s intervals for all trajectories.

The VACF quantifies the temporal correlation in the velocity of nanoparticles. The VACF is calculated as^43^

$$C_{v}\left(\tau \right)=\frac{\left\langle v\left(t\right)\cdot v\left(t+\tau \right)\right\rangle }{{\langle v\left(t\right)}^{2}\rangle }$$ 3

Here, **v**(*t*) and **v**(*t* + τ) are the particle velocities at time *t* and *t* + τ, respectively. The notation ⟨(·)⟩ denotes time average for a given trajectory. Figure 2d and e shows the PDF of displacements and ensemble VACF for each medium, respectively. Here, the VACF was calculated over each time frame (every 0.1 s) for individual trajectories. The ensemble VACF for each diffusion medium was then obtained by averaging the individual VACFs across all trajectories. For both 1.5 mM NaCl and 5 mM PBS, the displacement PDFs deviate from a Gaussian distribution, exhibiting long tails indicative of intermittent episodes of enhanced mobility. The corresponding VACFs approach zero but remain slightly anticorrelated in 1.5 mM NaCl.

AuNRs in H_2_O and 5 mM H_2_SO_4_ are likely exhibiting FBM-like subdiffusive dynamics, as evidenced by subdiffusive anomalous exponents (α < 1), displacement PDFs that remain close to Gaussian, and anticorrelated ensemble VACFs. In contrast, from the anomalous exponent values alone (α = 0.95 and α = 0.98), particles diffusing in 1.5 mM NaCl and 5 mM PBS are closest to exhibiting diffusive behavior near the Brownian limit (α → 1). However, the large scattering of the tMSD curves and the heavy-tailed PDF of displacements all indicate that the actual behavior is deviating from the normal Brownian limit,^45^ as Brownian motion is characterized by noncorrelated steps which are Gaussian distributed.^43^ These deviations from Gaussianity are hallmarks of weakly nonergodic, intermittent subdiffusion, often associated with continuous-time random walk (CTRW) or annealed transient time motion (ATTM) mechanisms of anomalous diffusion.^40,41,43,46–48^ In the context of diffusion, ergodicity is a property of processes in which the time-averaged and ensemble-averaged quantities converge over sufficiently long observation times. Physically, this implies that a particle undergoing ergodic diffusion uniformly explores its homogeneous environment, ensuring consistent behavior across individual trajectories. Conversely, nonergodic processes occur when this equivalence breaks down, causing substantial variations in individual particle trajectories. Physically, this arises because the particles become intermittently trapped, preventing full exploration of their heterogeneous environment. Consequently, this incomplete environmental sampling results in pronounced differences between individual trajectories and significant scattering in measured time-averaged properties.^43,48^

The dimensionless parameter, ξ, which is defined as the ratio of the tMSD calculated from an individual particle trajectory to the e-tMSD across the full population of trajectories, $\xi =\overset{―}{\delta r^{2}\left(\tau \right)}/\left\langle \overset{―}{\delta r^{2}\left(\tau \right)}\right\rangle$ , can quantify these deviations from ergodicity. The ergodicity breaking parameter (*EB*) further quantifies this variability, mathematically defined as the variance of the dimensionless parameter, ξ, calculated by *EB* = ⟨ξ^2^⟩ – 1. In this work, we evaluated ξ and the resulting EB value at the short-time delay limit (τ = 0.1 s) to compare ergodicity-breaking behavior across media.

Here, *EB* = 0 represents fully ergodic behavior, and increasingly large *EB* values indicate progressively stronger nonergodic dynamics.^43,48^ Given clear evidence of weakly nonergodic diffusion, we calculated *EB* for AuNRs diffusing in H_2_O, 5 mM H_2_SO_4_, 1.5 mM NaCl, and 5 mM PBS (see Figure S1). The corresponding *EB* values are 0.18, 0.23, 1.58, and 1.34, respectively. The *EB* values approaching 0 in the case of H_2_O and 5 mM H_2_SO_4_ indicate predominantly ergodic diffusion. In contrast, the significantly higher *EB* values observed in 1.5 mM NaCl and 5 mM PBS confirm the presence of weakly nonergodic behavior in these salt-containing environments.

Figures 1 and 2 show that changing the ionic composition of the liquid cell modulates both the magnitude and the mechanism of AuNR diffusion at the SiN_*x*_ interface. In H_2_O, and to a lesser extent in 5 mM H_2_SO_4_, AuNRs likely remain trapped by a combination of electrostatic and van der Waals interactions, and in the case of acidic solutions, hydrogen bonding, yielding confined, FBM-like dynamics. These dynamics are evidenced by subdiffusive short-time delay anomalous exponents and narrow spatial footprints (i.e., lower diffusion coefficients). In contrast, 1.5 mM NaCl and 5 mM PBS enable higher mobility, increasing the anomalous exponent and generalized diffusion coefficients (Figure 2), as seen in the e-tMSDs. Despite the increases in mobility observed in 1.5 mM NaCl and 5 mM PBS, the trajectories remain non-Brownian: heavy-tailed displacement PDFs, broad tMSD scatter, and nonzero ergodicity-breaking parameters all point to intermittent, weakly nonergodic dynamics consistent with CTRW or ATTM-type processes. The electrostatic screening in these cases likely weakens the electrostatic contribution to nanoparticle-surface coupling while maintaining residual nanoscale heterogeneity, thus promoting intermittent transitions between low- and high-mobility states, consistent with ATTM or CTRW-like intermittency. From a structural standpoint, the PEG ligand coating on the AuNRs is short (*M*_w_ = 1.63 kDa), forming a thin hydrated steric layer that can allow variations of the SiN_*x*_ membrane in each respective diffusion medium to modulate the particle-surface coupling.

From an interfacial electrochemistry standpoint, the Debye length (κ^–1^), which sets the range of the electrostatic component of the nanoparticle-surface interactions, shortens from ≈1000 nm in H_2_O to ≈2.9 nm in 5 mM H_2_SO_4_, ≈7.9 nm in 1.5 mM NaCl, and ≈4.0 nm in 5 mM PBS (see Table S3).^49^ As κ^–1^ decreases, long-range coupling weakens in all conditions, but medium-specific short-range interactions appear to dictate whether particles remain caged (FBM) or intermittently escape (ATTM/CTRW). In H_2_SO_4_, the screening would likely increase mobility by reducing the long-range electrostatic contribution, but low-pH protonation can strengthen PEG-surface hydrogen bonding. This short-range binding offsets the screening-driven mobility increase, such that the net effect is persistent FBM-like caging and mobility increases only modestly relative to H_2_O. This interpretation is supported by measured pH trends, where only 5 mM H_2_SO_4_ is strongly acidic (pH = 2.60) whereas H_2_O, 1.5 mM NaCl, and 5 mM PBS are near neutral (pH = 7.30, 7.03, and 7.73, respectively). In 1.5 mM NaCl, screening possibly acts without specific adsorption, yielding many effectively shallow interaction sites from which particles intermittently escape. In 5 mM PBS, electrostatic screening may reduce the effective depth of interfacial traps, while phosphate anions are expected to adsorb onto silanol groups via hydrogen bonding, passivating high-affinity adhesion sites and lowering the overall abundance of interfacial traps.^50^

Independent zeta potential (ζ) measurements are consistent with the above picture. PEG-AuNRs are near-neutral across all four media with ζ_AuNR_ values of −1.9 mV in H_2_O, +1.3 mV in 5 mM H_2_SO_4_, +1.2 mV in 1.5 mM NaCl, and −4.8 mV in 5 mM PBS (see Figure S2b). By contrast, the SiN_*x*_ surface potential reflects medium-dependent interfacial chemistry, with $\zeta_{{\mathrm{SiN}}_{x}}$ values of −41.0 mV in H_2_O, +14 mV in 5 mM H_2_SO_4_, −38 mV in 1.5 mM NaCl, and −11 mV in 5 mM PBS (see Figure S2a). The measured H_2_O $\zeta_{{\mathrm{SiN}}_{x}}$ is conducted under 0.5 mM KCl as a minimal background electrolyte required for accurate measurements. A positive surface zeta potential of the SiN_*x*_ membrane in 5 mM H_2_SO_4_ is consistent with surface protonation at low-pH acidic conditions. In contrast, 1.5 mM NaCl likely conducts electrostatic screening of the SiN_*x*_ membrane, while in 5 mM PBS, specific phosphate adsorption appears to reduce the magnitude of the negative surface potential. These medium-dependent interfacial characteristics are consistent with the observed transition from FBM-like confinement in H_2_O and 5 mM H_2_SO_4_ to intermittent, ATTM- or CTRW-type dynamics in 1.5 mM NaCl and 5 mM PBS. These ζ-potentials provide a bulk measure of how each medium reprograms the overall interfacial state, which can bias the distribution and characteristics of nanoscale heterogeneous interfacial interaction sites that ultimately govern the motion of PEG-AuNRs, despite their near-neutrality.

While the e-tMSDs reveal the subdiffusive nature of the translational diffusion of particles at the liquid–solid interface, a complete understanding of the motion of nanoparticles also requires characterizing rotational diffusion, which provides additional insight into how the interface restricts or enables orientational degrees of freedom. We next analyzed their in-plane rotational diffusion by characterizing the angular displacement, *δθ*, of AuNRs to assess how interfacial interactions influence orientational mobility across the same set of solution media. The corresponding analysis is shown in Figure 3.

**Figure 3:**
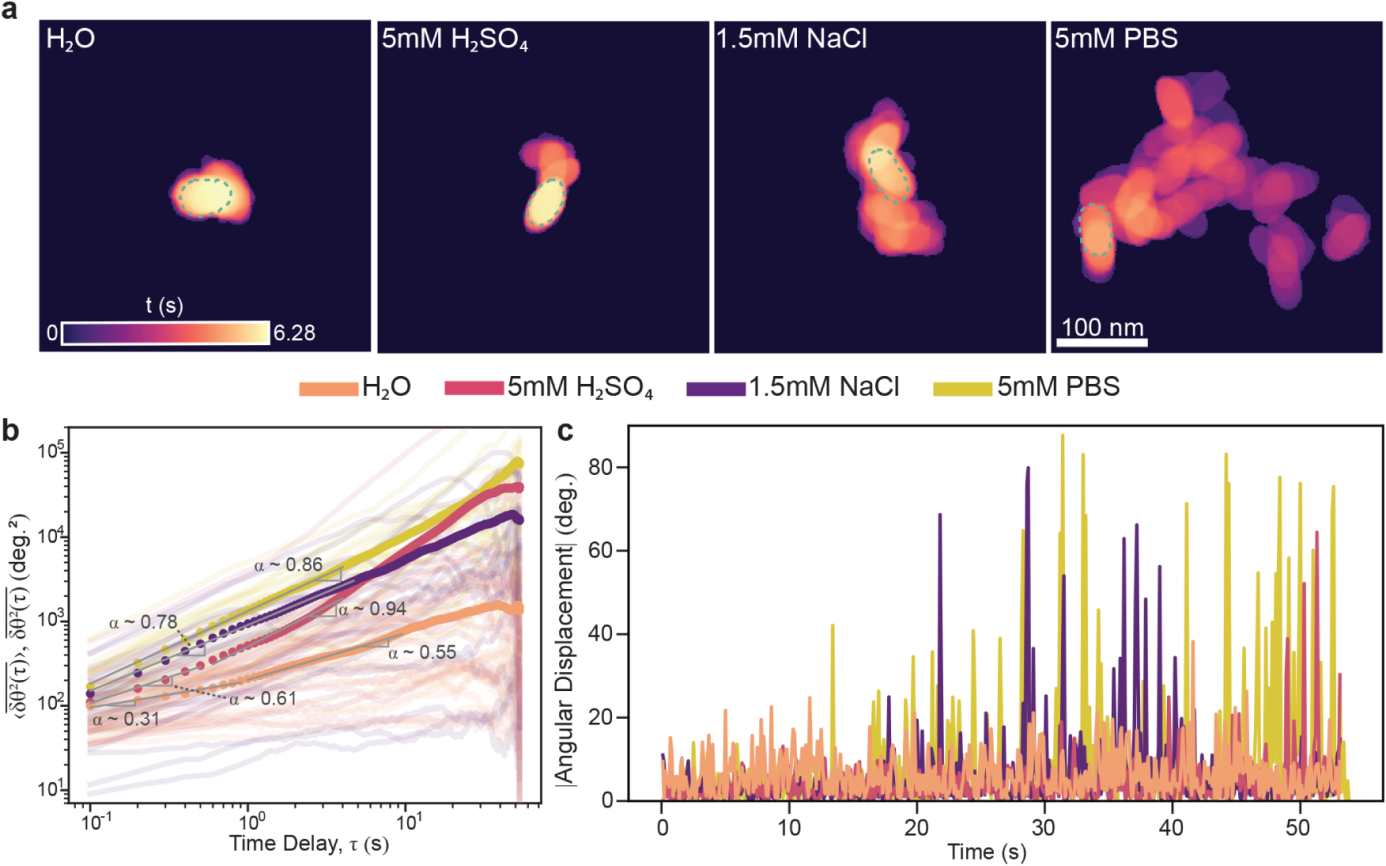
Statistical analysis of AuNR rotational diffusion in each respective solution. Colors are indicative of diffusion media: H_2_O (Orange), 5 mM H_2_SO_4_ (Pink), 1.5 mM NaCl (Purple), and 5 mM PBS (Yellow). (a) Residence-time heat maps showing particle position and orientation over time. The temporal scale bar is shown on a logarithmic scale. Dotted teal ellipsoids indicate the initial AuNR position. Scale bar applied to all conditions. (b) Ensemble-averaged time-averaged mean squared angular displacements (e-tMSADs) are shown with circular scatter points, overlaid on individual tMSADs as transparent solid lines. Power-law fits and angular anomalous exponents (α_θ_) are shown for each medium, including the short-time and long-time regimes shown separately when applicable. (c) Absolute angular displacement of a representative single AuNR trajectory from each solution.

Figure 3a shows the residence-time heat maps, highlighting how long a particle spends at any given position and orientation during the course of its trajectory. Each map was generated by summing the binarized pixel counts of segmented frames of the videos across time and converting them to real-time residence on a logarithmic scale.^26^ Particles diffusing in pure H_2_O show strong translational and orientational confinement, which is indicated by the long residence time and minimal rotation. Particles diffusing in 5 mM H_2_SO_4_ display slightly more rotational freedom; however, they still remain trapped in specific spots on the membrane surface over the course of the trajectory. Particles diffusing in 1.5 mM NaCl and 5 mM PBS exhibit the highest level of translational and rotational mobility. Among all cases, AuNRs in 5 mM PBS showcase the shortest residence times.

To quantify these observations, we calculated the ensemble-average of time-averaged mean squared angular displacement (e-tMSAD) for each condition (Figure 3b), defined as^26^

$$\left\langle \overset{―}{\delta \theta^{2}\left(\tau \right)}\right\rangle =\left\langle \overset{¯}{{\left(\theta_{t+\tau }-\theta_{t}\right)}^{2}}\right\rangle$$ 4

Here, θ represents the particle’s orientation angle, while *δθ* is the corresponding angular displacement. Prior to computing δθ­(τ), angular trajectories were corrected to account for discontinuities due to wrapping at ±180° using a piecewise unwrapping procedure (see for details).

The short- and long-time delay rotational anomalous exponents, α_θ_, were then extracted by fitting the e-tMSAD to a power law function, $\left\langle \overset{―}{\delta \theta^{2}\left(\tau \right)}\right\rangle \approx D_{\alpha_{\theta }}\tau^{\alpha_{\theta }}$ , as shown in Figure 3b.^43^ All rotational anomalous exponents were bootstrapped using the same procedure applied to the translational anomalous exponents extracted from e-tMSDs (see Table S2). Consistent with translational diffusion trends, AuNRs diffusing in 1.5 mM NaCl and 5 mM PBS do not show any crossover behavior in the e-tMSAD over time delays, having constant angular anomalous exponents of 0.78 (95% CI: 0.65–0.86) and 0.86 (95% CI: 0.80–0.92), respectively. In contrast, AuNRs diffusing in H_2_O and 5 mM H_2_SO_4_ do indeed present time-dependent orientational behavior: in H_2_O α_θ_ rises from 0.31 (95% CI: 0.18–0.45) at short time delays (<0.9 s) to 0.55 (95% CI: 0.37–0.69) at long delays, and in 5 mM H_2_SO_4_ α_θ_ shifts from 0.61 (95% CI: 0.46–0.71) at short time delays (<0.4 s) to 0.94 (95% CI: 0.60–1.11) at long time delays.

Interestingly, the short-time behavior of particles diffusing in H_2_SO_4_ exhibits a slightly larger short-time anomalous exponent for rotational motion compared to translational motion. The slightly higher angular mobility at short time delays in 5 mM H_2_SO_4_ can be rationalized by a “tip-pivot” mechanism, where the nanorod is anchored at one end but retains rotational freedom around that point, a behavior consistent with previously reported observations in LPTEM studies of nanorod dynamics.^26^ AuNR rotational mobility’s progressive increase from H_2_O to 5 mM H_2_SO_4_ to 1.5 mM NaCl to 5 mM PBS is also shown in Figure 3c, which plots the absolute values of the frame-to-frame (0.1 s) angular displacement over the course of a trajectory. The angular displacement of AuNRs in pure H_2_O remains flat and minimal over time, whereas those in 5 mM H_2_SO_4_ increase progressively, followed by greater angular displacements in 1.5 mM NaCl, and 5 mM PBS cases, indicating increasingly unconstrained rotational motion.

Collectively, Figures 2 and 3 demonstrate that the rotational and translational motion of AuNRs is possibly governed by shared interfacial interactions, where the ionic screening reduces the electrostatic contribution to the net near-surface interaction potential, decreasing confinement, and allowing both translational and orientational mobility. This is further supported by the increasing correlation between radial and angular displacements across H_2_O, 5 mM H_2_SO_4_, 1.5 mM NaCl, and 5 mM PBS (see Figure S3). The progressive increase in correlation suggests that as interfacial screening increases, translational and rotational motion become increasingly coupled, marking a transition from localized pivoting or trapped behavior to a more coordinated displacement. This reflects a simultaneous reduction in energetic barriers to both modes of motion, resulting in enhanced overall nanoparticle mobility. Notably, the lower correlation observed in H_2_O and 5 mM H_2_SO_4_, which are conditions that display FBM-like characteristics, is consistent with prior observations in complex fluids, where translational and rotational dynamics may exhibit nontrivial behaviors.^51^

### Anomalous Diffusion Classification

Given the signatures of varying classes of anomalous subdiffusion observed in our translational and orientational analyses, we applied a deep learning-based anomalous diffusion classifier, MoNet 2.0,^32,37^ to identify the underlying class of diffusion exhibited by AuNRs within each solution condition (see SI and Methods for details on MoNet 2.0). The classification results for all 20 translational trajectories in each solution condition are shown in Figure 4 as the predicted probability of each class of diffusion for a given trajectory. Trajectories from H_2_O and 5 mM H_2_SO_4_ cases are classified with an average probability of 80% and 76.5% for FBM, respectively. In 1.5 mM NaCl and 5 mM PBS, the dominant class shifts decisively: MoNet 2.0 assigns average probabilities of 85.0% and 99.8% for ATTM, respectively. A one-sample *t*-test against a uniform distribution (null hypothesis: 16.7% for each of the six classes) yielded *p*-values< 0.0001 for all 4 conditions, confirming the statistical significance of these assignments.

**Figure 4:**
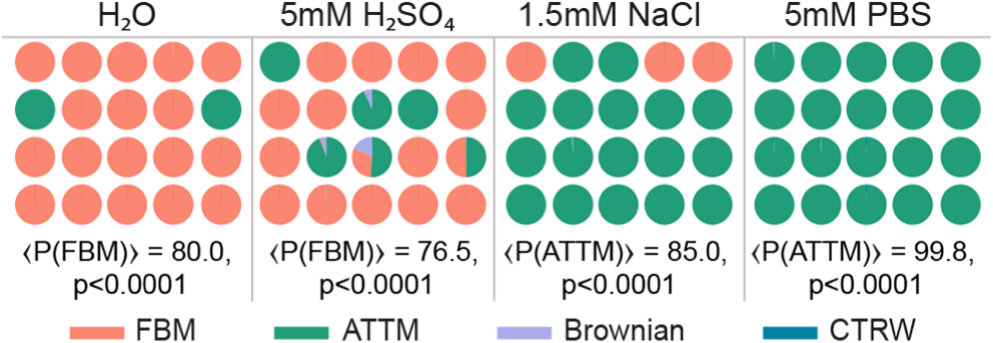
MoNet 2.0 classification of 20 translational AuNR trajectories recorded near the SiN*_x_* membrane in each diffusion medium (H_2_O, 5 mM H_2_SO_4_, 1.5 mM NaCl, 5 mM PBS). Pie charts display the probabilities for the six diffusion classes: Brownian motion (BM), fractional Brownian motion (FBM), continuous-time random walk (CTRW), annealed transient time motion (ATTM), Lévy walk (LW), and scaled Brownian motion (SBM) returned by MoNet 2.0. The legend displays only the four diffusion classes that MoNet 2.0 assigned nonzero probability in this data set (FBM, ATTM, Brownian, CTRW). Beneath each medium condition, the dominant class’s average predicted probability and the associated *p*-value from a one-sample *t*-test (against a uniform null of 16.7%) are reported.

These classification results reinforce our earlier canonical statistical analyses conducted in the previous section, confirming that ionic screening steers nanorod motion from viscoelasticity-driven FBM, characteristic of confined motion in H_2_O and 5 mM H_2_SO_4_, to intermittent, weakly nonergodic time-dependent-diffusivity-driven ATTM in salt-containing solutions.

### Viscoelastic Modeling of Surface Diffusion

The identification of anomalous diffusion classes across the different ionic environments suggests a complex interplay of interactions at the liquid–solid interface in the LPTEM cell. Collapsing the e-tMSDs for FBM-classified trajectories from H_2_O and 5 mM H_2_SO_4_ yields a single master curve (see Figure S4) that confirms similar subdiffusive scaling expected for viscoelasticity-driven FBM.^52–54^

To quantify the interfacial mechanical response underlying the observed subdiffusion, we conducted passive nanorheology using the resulting translational and rotational particle trajectories in H_2_O and 5 mM H_2_SO_4_. This approach infers the effective mechanical response experienced by the nanoparticles, arising from the combination of surface interaction forces and viscous dissipation in the confined liquid layer near the substrate, directly from their motion. Specifically, we employed a nanorheology framework based on the frequency-dependent generalized Stokes–Einstein relation (GSER)^52–57^ to reconstruct the corresponding viscoelastic response across multiple time scales. Thus, passive nanorheology provides an effective viscoelastic description of nanoparticle-surface interactions.

We define *G̃*(*s*) as the Laplace-space complex shear modulus that describes the effective mechanical response of the interfacial environment experienced by the AuNRs. Within the GSER framework, the fluctuation–dissipation theorem connects the ensemble time-averaged mean-squared displacement (e-tMSD) of a thermally driven probe to *G̃*(*s*), enabling us to infer viscoelastic moduli directly from the measured trajectories. Therefore, we first computed the Laplace-space complex modulus, *G̃*(*s*), from the e-tMSDs using a gamma function (Γ)-based approximation:

5 $$\mathrm{G̃}\left(s\right)\approx \frac{k_{B}T}{\pi a\left\langle \overset{―}{\delta r^{2}\left(\tau \right)}\right\rangle \Gamma \left[1+\frac{\partial \ln\left\langle \overset{―}{\delta r^{2}\left(\tau \right)}\right\rangle }{\partial \ln\tau }\right]}{|}_{\tau =1/s}$$

For rotational passive rheology, we replaced the translational e-tMSD by the e-tMSAD, and used the rotational GSER.^58^ In Laplace space, we estimated the modulus using the same form as the translational GSER but with a rotational prefactor:

6 $${\mathrm{G̃}}_{\theta }\left(s\right)\approx \frac{k_{B}T}{C_{\mathrm{rot}}\left\langle \overset{―}{\delta \theta^{2}\left(\tau \right)}\right\rangle \Gamma \left[1+\frac{\partial \ln\left\langle \overset{―}{\delta \theta^{2}\left(\tau \right)}\right\rangle }{\partial \ln\tau }\right]}{|}_{\tau =1/s}$$

where *C*_rot_ is the rotational geometry constant for our nonspherical probe. Specifically, *C*_rot_ generalizes the spherical rotational GSER prefactor 4*a* ^3^ to rods by *C*_rot_ = 4*a*_eq_^3^ *S*_rot_ where *a*_eq_ is the equal-volume spherical radius and *S*_rot_ is a dimensionless correction factor proportional to the ratio of the particle’s rotational diffusion coefficient to that of a same-radius sphere.^59,60^ Here, *k*_B_ is the Boltzmann constant, *T* is the temperature, *a* is the effective hydrodynamic radius of the particle, approximated as 37.5 nm (half of the AuNR length, which is 75 nm) to simplify the Stokes conversion. Since this radius only contributes as a scalar prefactor, it does not affect the relative trends in the moduli obtained. All calculations were performed at *T* = 298 K and constant electron flux of 30 e^–^/(Å^2^·s) for all trajectories. To ensure analysis across consistent irradiation exposure and minimize noise at long time delays, we applied this framework to the first 200 frames of each e-tMSD and e-tMSAD.

The relaxation spectra were then discretized using non-negative least-squares fitting to find *G*_*j*_, which represents the magnitude of each discrete relaxation mode, *j*, and the corresponding relaxation times, τ_*j*_.

7 $$\mathrm{G̃}\left(s\right)\approx \underset{j}{\sum }\frac{G_{j}s}{s+1/\tau_{j}}$$

From the fitted weights and relaxation times, we reconstructed the viscoelastic moduli, *G*′(ω) and *G*″(ω), using standard Fourier transforms.

8 $$G^{\prime }\left(\omega \right)=\underset{j}{\sum }\frac{G_{j}{\left(\omega \tau_{j}\right)}^{2}}{1+\omega^{2}\tau_{j}^{2}},,G^{\prime \prime }\left(\omega \right)=\underset{j}{\sum }\frac{G_{j}\omega \tau_{j}}{1+\omega^{2}\tau_{j}^{2}}$$

Here, *G*′(ω) represents the storage modulus, capturing the elastic or solid-like response, while *G*″(ω) denotes the loss modulus, reflecting the viscous or energy-dissipative behavior of the material.^39^ In the context of AuNR surface diffusion at the SiN_*x*_ interface, these moduli describe the effective viscoelasticity arising from the interfacial interaction landscape, where the elastic contribution reflects the restoring forces imposed by nanoparticle-surface potentials, while the viscous component originates from the energy dissipation in the confined liquid layer and thermally activated fluctuations.

The resulting viscoelastic moduli for each respective diffusion medium are shown in Figure 5a–d. Here, we plotted the moduli as functions of linear frequency *f* = ω/2π (Hz) to align the axes with the acquisition bandwidth. We marked the Nyquist frequency (5 Hz = 1/(2 × 0.1 s), where 0.1 s is the sampling interval) on each modulus plot to denote the highest frequency that can be reliably reconstructed from the trajectory data. In all cases, the crossover points fall below this limit, ensuring that the reported moduli correspond to physically valid regimes. For translationally and rotationally derived moduli from H_2_O and 5 mM H_2_SO_4_, the storage modulus is dominant at high frequencies (short time delays), followed by a crossover to a loss modulus-dominated regime at low frequencies (long time delays). This crossover frequency (*f*_*c*_) marks the transition from elastic to viscous behavior and is a key signature of viscoelasticity. The relaxation time (τ_*c*_ = 1/(2*πf*_*c*_)) and the crossover moduli values for each solution vary as shown in Figure 5e–h with 95% confidence intervals estimated by bootstrapping (2000 resamples with replacement) of the crossover moduli and relaxation times (see Table S4 and Table S5).

**Figure 5:**
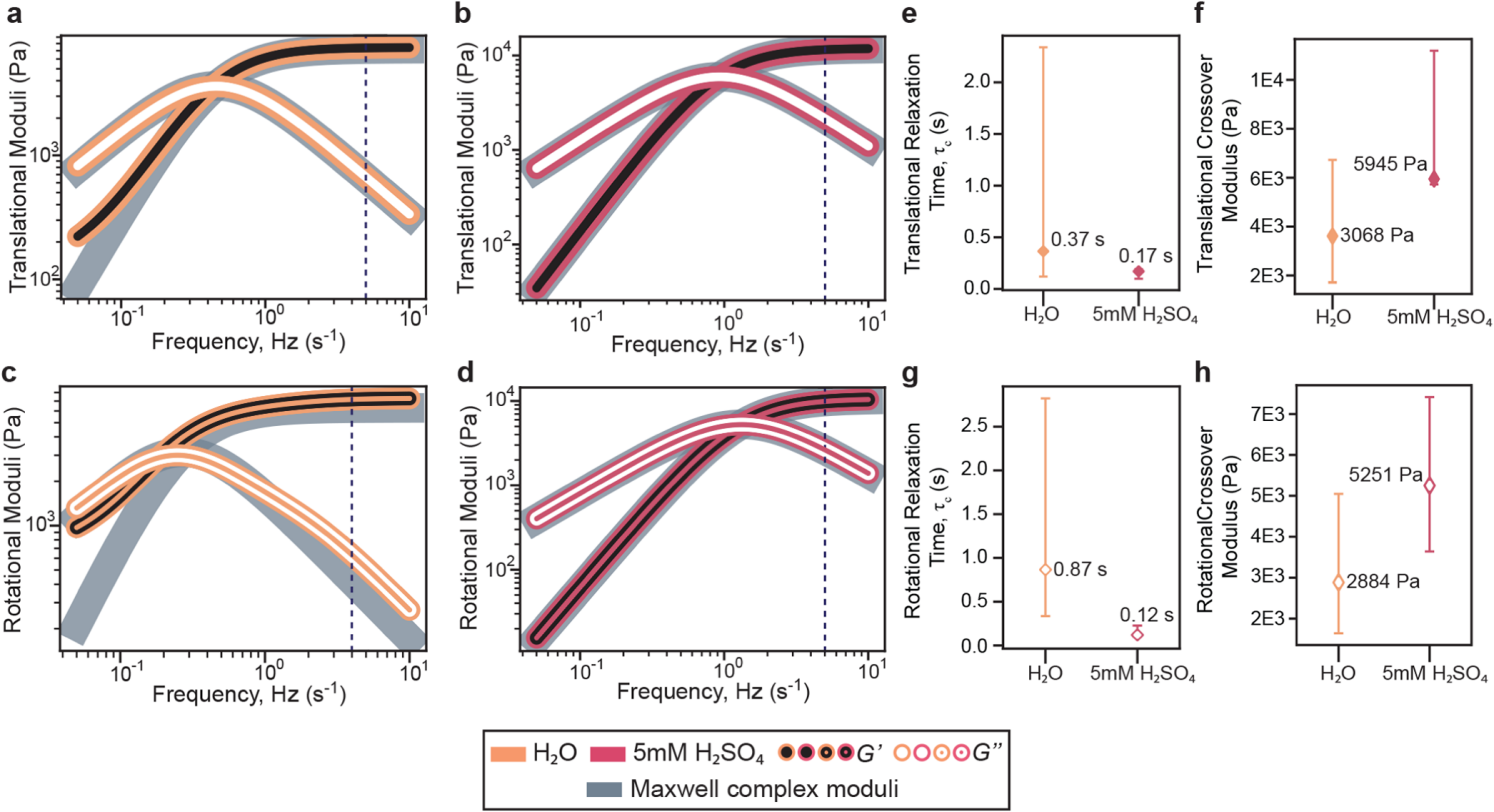
Nanorheology in LPTEM: effective viscoelastic response of the near-surface environment probed by AuNR translational and rotational diffusion near the SiN*_x_* membrane. Translational (a,b) and rotational (c,d) frequency-dependent storage (*G*′, black markers) and loss (*G*″, white markers) moduli obtained from translational e-tMSDs and rotational e-tMSADs. Marker color indicates the solution medium: H_2_O (orange) and 5 mM H_2_SO_4_ (pink), denoting the elastic and viscous components of the interfacial response, respectively. Gray curves represent fits to a single-relaxation-time Maxwell model. The Nyquist frequency for the sampling interval (*δτ* = 0.1 s) is 5 Hz and is marked by a dashed line. (e, g) Characteristic relaxation times (τ*_c_*) and (f,h) crossover moduli (*G_c_*) for each medium; white diamonds indicate values derived from rotational (e-tMSAD-based) moduli. Error bars represent 95% confidence intervals from 2,000 bootstrap resamplings with replacement.

Across translational and rotational analyses, 5 mM H_2_SO_4_ exhibits faster relaxation times than H_2_O, shown in Figure 5e–h: the translational and rotational relaxation times are 0.17 s and 0.12 s in H_2_SO_4_, compared with 0.37 s and 0.87 s in H_2_O. The corresponding crossover moduli are higher in H_2_SO_4_ (5945 and 5251 Pa) than in H_2_O (3068 and 2884 Pa), likely resulting from both the higher effective viscosity of H_2_SO_4_ and additional hydrogen bonding between the protonated SiN_*x*_ surface at low pH and PEG ligands on the AuNRs.^61^ We then fit the effective viscoelastic moduli to a single-relaxation-time Maxwell fluid model^52,62,63^ as shown in Figure 5a–d. Both the translational and rotational moduli are well described by a single-relaxation-time Maxwell-type response. At high frequencies (short time delays), the particle experiences predominantly elastic restoring forces from the surface interaction potentials, whereas at low frequencies (long time delays), the response is dominated by viscous dissipation in the confined liquid layer (see Figure S5).

To further support the validity of the single-relaxation-time Maxwell framework, we modeled the ensemble-averaged mean-squared displacement (e-tMSD) expected for a particle diffusing in an effective viscoelastic Maxwell medium following established theoretical formulations.^52^ The resulting expressions were then fit to the experimentally obtained e-tMSDs for each diffusing condition (see Figure S6), yielding excellent agreement with the experimental data (*R* ^2^ = 0.99). This consistency supports the Maxwell model as an effective approximation for describing the response sensed by the nanoparticles in the liquid cell environment.

Collectively, the rheological reconstructions from translational and rotational AuNR trajectories near the SiN_*x*_ membrane under FBM-classified conditions (H_2_O and 5 mM H_2_SO_4_) suggest that the effective interfacial mechanical response is well captured by a single-relaxation-time Maxwell model, where the ionic environment appears to tune the effective viscoelastic parameters, i.e., the relaxation time (τ_*c*_) and crossover modulus, *G*_*c*_. Within this framework, local interaction sites act as effective viscoelastic traps, where the elasticity reflects the stiffness of the nanoparticle-surface interaction potential that transiently confines the nanoparticle, while the viscosity arises from the dissipation associated with thermally activated fluctuations and hops within and between traps. In ion-poor H_2_O, a collection of electrostatic and van der Waals interactions likely sustains elastic confinement over longer time scales, yielding a slower elastic-to-viscous transition. In 5 mM H_2_SO_4_, the increased screening shortens the Debye length and weakens the long-range electrostatic contribution to nanoparticle-surface coupling, which can increase mobility and contribute to faster relaxation. Even with a shorter Debye length, the sustained FBM-like confinement is consistent with the additional H-bonding arising from protonation of the SiN_*x*_ membrane at low pH and with a possible increase in viscosity, both of which can contribute to the higher elastic-to-viscous crossover modulus values observed in 5 mM H_2_SO_4_ compared to H_2_O case.^61^

Overall, under FBM-classified conditions, the interface exhibits an effective single-relaxation-time Maxwell response. Mapping the frequency-dependent moduli to the statistics of caged motion links interfacial mechanics to stochastic transport. In this framework, the relaxation time specifies the time scale for the elastic-to-viscous transition, and the crossover modulus reports the effective cage stiffness. Together, these parameters suggest how protonation of surface silanol groups, and the resulting hydrogen bonding to PEG, modulate the effective particle–membrane interaction potential, increasing the stiffness of PEG-surface binding sites. Notably, both translational and rotational MSD analyses yield consistent relaxation times and crossover moduli in H_2_O and 5 mM H_2_SO_4_, supporting the applicability of the Maxwell model for describing FBM-like confinement at the SiN_*x*_ interface. Consequently, by tuning solution condition and thus the SiN_*x*_ membrane’s surface chemistry, one can predictably shift the relaxation time and the crossover modulus and, in turn, modulate confinement versus release of particle transport at the liquid cell membrane. This effective viscoelastic framework provides practical design principles for controlling nanoparticle mobility in LPTEM.

### Discussion

The anomalous diffusion of PEG-coated AuNRs near the SiN_*x*_ membrane in pure H_2_O arises from a complex interplay of electrostatic and van der Waals interactions at the heterogeneous surface interaction sites (SiO^–^, ${\mathrm{SiNH}}_{2}^{+}$ ).^20^ These forces result in a spatially heterogeneous interfacial potential energy landscape with localized interaction wells that may locally exceed thermal energy, yielding non-Brownian dynamics of nanoparticles diffusing near the interface.^32^ In pure H_2_O, this balance produces strong caging and reduced mobility, leading to FBM-like dynamics, as evidenced by sublinear e-tMSDs, Gaussian displacement PDFs, and anticorrelated VACFs (see Figure 2).

To systematically modulate these interactions, we employed three solution chemistries with distinct interfacial actions: (1) 5 mM H_2_SO_4_ to lower pH and protonate silanol groups, (2) 1.5 mM NaCl to introduce monovalent electrostatic screening, and (3) 5 mM PBS to combine electrostatic screening with phosphate adsorption. Ex situ zeta-potential measurements and Debye-length calculations (see Figure S2 and Table S3) confirm that these media systematically alter the SiN_*x*_ surface charge while leaving PEG-AuNRs nearly neutral. Together, these measurements and calculations verify that the chosen solution chemistries reshape the membrane’s interfacial potential landscape rather than modifying the particle charge, thereby modulating AuNR-surface interaction strength and the resulting diffusion mechanism.

In 5 mM H_2_SO_4_, moderate increases in anomalous exponents (α) are consistent with screening that reduces the long-range electrostatic contribution to the nanoparticle-surface interaction landscape, which would increase mobility. In parallel, low-pH silanol protonation strengthens PEG-surface hydrogen bonding and maintains short-range adhesion. These opposing effects are partially offsetting, producing only brief release events and predominantly FBM-like confinement. In contrast, 1.5 mM NaCl enhanced screening may substantially reduce the effective depth of surface traps, allowing more frequent escape events that manifest as heavy-tailed displacement PDFs, a hallmark of ATTM-type intermittent motion. In 5 mM PBS, mobility increases further and ATTM predominates. In this case, phosphate adsorption potentially passivates high-affinity interaction sites while mono- and multivalent ions further contribute to electrostatic screening, decreasing the abundance and the strength of trapping (interaction) sites. Consequently, PEG-coated AuNRs traverse larger distances before encountering another trapping site, leading to higher overall mobility (Figures 2 and 3).

It is important to note that the solution chemistry could influence the PEG hydration and solubility. However, the concentrations used here are low-mM, well below regimes typically associated with pronounced structural changes.^64–67^ Consistent with this, the PEG-AuNRs in our system remain colloidally stable during imaging (no observable aggregation or irreversible morphology/contrast changes), and all reported statistics are computed from spatially isolated single-particle trajectories. Therefore, solution chemistry-dependent diffusion trends are interpreted primarily as medium-dependent changes in particle-SiN_*x*_ coupling at a heterogeneous interface rather than changes in the PEG layer itself.

Electron irradiation of aqueous media inevitably generates a suite of radiolysis species (e.g., H^+^, OH^–^) that reach steady-state concentrations within microsecond to millisecond time scales.^68–71^ In pure water, these species may modestly shorten the effective Debye length, whereas in 5 mM H_2_SO_4_, 1.5 mM NaCl, and 5 mM PBS, the bulk charged species likely already compress the Debye length by approximately 3 orders of magnitude (see Table S3). Therefore, any additional screening from radiolysis species is expected to be a comparatively small correction in these latter conditions. We acknowledge that the addition of H_2_SO_4_, NaCl, or PBS can steer the underlying radiolysis reaction network in composition-dependent ways, but a detailed kinetic treatment is beyond the scope of this work. Instead, we focus on how the preset ionic environment controls the diffusion class at the interface, and we ground this interpretation in Debye-length estimates and ex situ zeta-potential measurements (see Figure S2). Within this framework, radiolysis may act as a secondary perturbation, but it is the solution composition that is the primary control for the observed changes in single-particle diffusion behavior at the SiN_*x*_ membrane. Charging of the SiN_*x*_ windows can produce localized positive charge centers and low-energy secondary electrons that enhance near-window radiolysis. However, with identical irradiation parameters (accelerating voltage, electron flux, beam radius, and acquisition rate) across all media, such charging effects are expected to remain effectively similar. Finally, although PEG polymer ligands can undergo beam-induced cross-linking or scission^72^ on longer time scales, which can also be influenced by solution-chemistry-dependent radiolysis pathways, our ≤ 60 s acquisitions show no irreversible changes in contrast, morphology, or aggregation in all four solution chemistry conditions studied. The strong correlation between mobility and initial ionic environment, therefore, supports solution-controlled modulation of particle-interface interactions as the dominant mechanism, as opposed to beam-induced polymer modification. Together, these considerations justify attributing the media-dependent diffusion behavior to the solution-controlled interfacial potential landscape, rather than to electron-beam-induced artifacts.

Deep learning classification results from MoNet 2.0 further corroborate this mechanistic picture, where FBM dominates under strong confinement (H_2_O and 5 mM H_2_SO_4_), while ATTM is predominant in screened environments (1.5 mM NaCl and 5 mM PBS) (Figure 4). These results suggest that FBM corresponds to persistent viscoelastic caging imposed by surface interaction potentials, while ATTM reflects a weakly nonergodic regime in which nanoparticles remain mobile but experience transient slowdowns^40,41,48^ rather than static immobilization. This contrasts with CTRW-like models that assume discrete trapping events and were previously associated with subdiffusion of AuNRs in LPTEM at high electron fluxes.^11–29,31,32^ In the ATTM regime, particles traverse an interaction potential landscape with shallow potential wells and localized adhesion sites that enable transitions between low- and high-mobility states, resulting in heavy-tailed displacement PDFs and diminished VACF anticorrelation, characteristics of transiently heterogeneous diffusivity.

To evaluate whether the solution-chemistry-dependent mobility trends are specific to the near-neutral PEG-AuNRs, we performed additional experiments using cationic CTAB-coated AuNRs in H_2_O and 5 mM PBS, analyzed with the same tracking and e-tMSD workflow (see Figure S7 and Table S6). CTAB-AuNRs in H_2_O and 5 mM PBS showcase subdiffusive power-law e-tMSD scaling in both media but increased mobility in 5 mM PBS, indicating that the mobility enhancement is not PEG-specific and is consistent with solution chemistry tuning of particle-SiN_*x*_ coupling.

To further interrogate the mechanical nature of confinement, we conducted passive nanorheology using the e-tMSDs and e-tMSADs of FBM-classified media (H_2_O and 5 mM H_2_SO_4_). Both translationally and rotationally derived viscoelastic moduli exhibit a transition from predominantly elastic behavior at short time delays to viscous dissipation at longer time delays (Figure 5a–d). These experimentally derived viscoelastic moduli reflect the balance between confinement elasticity and dissipative relaxation within the near-surface environment. Rheological model analysis (Figure S5) confirms that the interfacial response is well described by a single-relaxation-time Maxwell model, validating this framework as a compact physical representation of the effective interfacial mechanical behavior sensed by the nanoparticle.

Notably, 5 mM H_2_SO_4_ exhibits a shorter relaxation time than H_2_O, consistent with the reduced Debye length arising from higher ionic strength, which likely suppresses long-range electrostatic contributions to the nanoparticle-surface interaction landscape. In this screened landscape, thermally activated fluctuations can more efficiently dissipate stored elastic energy, leading to faster stress relaxation. At the same time, protonation of the SiN_*x*_ membrane introduces PEG-surface hydrogen bonding that sustains caging and increases the local stiffness of interaction sites, yielding a higher elastic-viscous crossover modulus value than in H_2_O. The long Debye length in H_2_O likely maintains electrostatic coupling between AuNRs and the SiN_*x*_ membrane, suggesting that confinement persists over longer time scales, making effective escape from the caged regime less frequent and slowing the elastic-to-viscous transition.

This framework suggests that local interaction sites act as transient viscoelastic traps, where the effective elasticity captures the stiffness associated with intermittent surface confinement and the effective viscosity reflects dissipation likely arising from internal rearrangements and thermally activated fluctuations and hops within and between adhesive sites. At short time delays, particles remain largely confined within these traps, so the response is dominated by the elastic contribution. At longer time delays, thermal fluctuations enable repeated release from these traps often enough that viscous dissipation becomes the prevailing response. Importantly, translational and rotational analyses yield nearly consistent relaxation times and crossover moduli in both H_2_O and 5 mM H_2_SO_4_, underscoring the robustness of this Maxwell description under FBM-classified conditions. Collectively, these results also demonstrate that passive nanorheology provides a direct experimental probe of effective nanoscale interfacial mechanics, offering quantitative means for interpreting dynamics and mechanical response at a spatial scale specifically accessible by LPTEM.

The findings of this work establish that anomalous diffusion of nanoparticles at the liquid–solid interface in LPTEM is governed not only by particle properties but also by the tunable interfacial landscape defined by ionic composition and the resulting interfacial interactions. Under FBM-classified conditions, the effective Maxwell description connects stochastic transport statistics to interfacial mechanical response, providing quantitative readouts of confinement strength and thermally driven release. More broadly, these findings show that solution chemistry (pH, ionic strength, and specific ion effects) can be used to tune both the mobility and anomalous diffusion mechanism of near-surface nanoparticles, offering mechanistic insight into particle-interface coupling in LPTEM.

## Conclusions

Overall, this study establishes a mechanistic understanding of the anomalous diffusion at the liquid–solid interface in the liquid-cell of LPTEM, advancing its role from a visualization tool to a predictive framework for SPT and a quantitative platform for passive nanorheology. Our results demonstrate that nanoparticle transport at the liquid–solid interface in LPTEM is governed by a tunable interfacial potential landscape, where variations in ionic composition and the resulting interfacial interactions control the balance between confinement and mobility. In H_2_O and 5 mM H_2_SO_4_, long-range electrostatic coupling and hydrogen bonding result in FBM-like confinement, while increased ionic screening in 1.5 mM NaCl and phosphate adsorption in 5 mM PBS can progressively lower the abundance and strength of interaction sites, leading to ATTM-like intermittent motion with higher mobility. These results demonstrate that solution composition provides an external handle for controllably tuning anomalous transport at the nanoscale, establishing a predictive link between interfacial chemistry and nanoparticle dynamics in LPTEM.

To directly probe the mechanics underlying these dynamics, we applied passive nanorheology to translational and rotational trajectories of FBM-classified media. The experimentally derived effective viscoelastic moduli show an elastic-dominated response at short time delays that gives way to viscous dissipation at longer time delays. Model analysis supports a single-relaxation-time Maxwell description in both media. The shorter relaxation time in H_2_SO_4_ can be a reflection of the reduced Debye length, while the higher crossover modulus suggests added effective elasticity from PEG-surface hydrogen bonding. Within this framework, the elastic component reflects resistance arising from the stiffness of intermittent surface-confinement potentials, and the viscous component reflects dissipation of energy as particles are released from adhesive sites. The agreement between translationally and rotationally derived relaxation times and crossover moduli in both H_2_O and H_2_SO_4_ underscores the robustness of this viscoelastic interpretation and establishes passive nanorheology as a quantitative probe of interfacial dynamics in operando electron microscopy.

Together, these findings transform LPTEM from a qualitative visualization tool to a quantitative platform for probing effective nanoscale mechanical landscapes and predicting particle transport in complex environments. By delineating how solution chemistry and the resulting surface interactions govern near-surface mobility, this work introduces experimentally accessible levers, including electrolyte composition, pH, and interfacial passivation, for systematically controlling nanoparticle mobility at the interface. These capabilities provide general design principles for placing nanomaterials in targeted transport regimes in a multitude of research domains, from steering particle motion at catalytic interfaces to regulating transport across biomembranes and controlling mobility in phase-separated or polymeric environments where interfacial chemistry evolves over time. Therefore, this study not only reveals mechanisms of anomalous diffusion at the SiN_*x*_ membrane in LPTEM but also establishes a framework that enables predictive and tunable control over nanoscale transport at liquid–solid interfaces.

## Methods

### Materials

Polyethylene glycol (PEG) coated AuNRs (catalog no. RFL-25–700) were purchased from CD Bioparticles. Sulfuric acid (H_2_SO_4_, 95–98%) was purchased from VWR Chemicals. Sodium chloride (NaCl, >99.0%) was purchased from Sigma-Aldrich. Phosphate Buffered Saline (PBS) was purchased from Ward’s Science. Deionized water (DI water, Millipore) was used for all aqueous solutions. All chemicals were used without further purification.

### LPTEM Experiments

Commercially available silicon nitride microchips (Protochips Inc.) were used for imaging particle surface diffusion in LPTEM. For all solutions, liquid cell top chips (catalog no. EPT-52W-10) and bottom chips (catalog no. EPB-52DNS-10) with a static spacer size of 150 nm were used. All chips were given a 2 min immersion in acetone to remove the photoresist coating and subsequently immersed in ethanol for another 2 min. Excess ethanol was blotted off with filter paper and the chips were further dried by flowing N_2_ gas across the surface. Following the cleaning, the chips were surface treated by glow discharge in a PELCO easiGlow system at 0.39 mbar, 15 mA for 45 s to render the surfaces hydrophilic and increase wettability. The chips were then assembled into a Poseidon Select holder with 0.75 μL of liquid solution. *In situ* experiments were conducted on a FEI Tecnai F30 TEM with a thermal field emission gun operating at 200 kV in the Materials Characterization Facility of the Institute for Matter and Systems at Georgia Institute of Technology. Videos of the AuNR surface diffusion were collected using a Gatan Oneview camera and recorded using the Digital Micrograph software. All experimental data were collected at a magnification of 19.5kx, 1024 × 1024 pixels, 100 frames per second (fps) (binned to 10 fps postacquisition), an electron flux of 30 e^–^/(Å^2^ · s), and an approximate beam radius of 2.5 μm.

### Particle Trajectory Segmentation and Tracking

Postacquisition, images are binned to 10 frames per second using average pixel binning, increasing the signal-to-noise ratio to ensure accurate image segmentation and analysis. Images are segmented and particle trajectories are tracked using SAM-EM,^42^ a foundation model for segmentation and tracking, fine-tuned on simulated LPTEM videos. The top 10 longest trajectories were selected from each duplicate acquisition for every solution condition, yielding 20 trajectories per medium. The shortest trajectory length across all conditions was 532 points; therefore, all trajectories in each condition were uniformly truncated to this length for subsequent analysis.

### Diffusion Classification Using MoNet 2.0

To classify anomalous diffusion mechanisms from particle trajectories, we adapted MoNet 2.0, a deep learning model based on dilated convolutional neural networks. The original MoNet architecture was designed to classify three diffusion classes: Brownian motion (BM), fractional Brownian motion (FBM), and continuous-time random walk (CTRW), using 300-frame trajectories.^32^ In previous work,^37^ MoNet was extended into MoNet 2.0 to support the classification of seven diffusion classes, including experimental LPTEM trajectories. In this study, we retrained MoNet 2.0 to classify among six diffusion classes: BM, FBM, CTRW, annealed transient time motion (ATTM), scaled Brownian motion (SBM), and Lévy walk (LW), using 532-frame trajectories. Each input trajectory is converted to its frame-to-frame displacements, which are input into MoNet 2.0.

MoNet 2.0 processes each trajectory through seven parallel branches of 1D dilated convolutional layers with varying kernel sizes and dilation rates. Each branch includes three convolutional layers with causal padding and ReLU activation, followed by batch normalization and global max pooling. The extracted features are concatenated and passed through fully connected layers with 512 and 128 units, followed by a softmax output layer returning the predicted probability across the six diffusion classes.

The model is trained on a simulated data set of 48,000 2D trajectories (8,000 per class), generated using the publicly available simulation code provided by the Anomalous Diffusion (AnDi) challenge^48^ without any additional noise. All simulated trajectories are normalized prior to training by shifting each coordinate to start at zero and scaling both *x* and *y* by the global range across both dimensions. The resulting trajectories are then converted to frame-to-frame displacements before being input to the model. Training is conducted using the Adam optimizer with a learning rate of 10^–3^ for 20 epochs. The final model performance is evaluated on an independent test set of 4,800 trajectories. A macro-averaged F1 score (identical to the weighted F1 score due to class balance) of 0.96 is achieved, indicating high classification accuracy across all six diffusion classes.

### Surface ζ-Potential Measurements of SiN*_x_* Membrane

The ζ-potential measurements of SiN_*x*_ membrane used a Zetasizer Nano ZS (Malvern Panalytical) via phase analysis light scattering and the Smoluchowski approximation. SiN_*x*_ chips were treated by glow discharge in a PELCO easiGlow system at 0.39 mbar, 15 mA for 45 s, following the same procedure used for the LPTEM experiments, then immersed in each medium (H_2_O, 5 mM H_2_SO_4_, 1.5 mM NaCl, 5 mM PBS). Standard 200 nm polystyrene tracer beads (Malvern) were dispersed in the corresponding medium and used to probe the near-surface electro-osmotic mobility. Measurements were performed at 25^◦^C in triplicate for each condition. ζ-potentials were calculated from the measured electrokinetic mobility using the Smoluchowski relation and are reported as mean ± one standard deviation in the Supporting Information.

### ζ-Potential Measurements of PEG-AuNR Dispersions

The ζ-potential measurements of PEG-coated gold nanorods were measured in each medium (H_2_O, 5 mM H_2_SO_4_, 1.5 mM NaCl, 5 mM PBS) using a Zetasizer Nano ZS (Malvern Panalytical) via phase analysis light scattering and the Smoluchowski approximation. Dispersions were prepared by 1:10 dilution in each respective medium, exactly as used in the LPTEM experiments. Measurements were performed in triplicate using three independently prepared samples per medium. ζ-potentials were computed from electrophoretic mobility and are reported as mean ± one standard deviation in the Supporting Information.

### Molecular-Weight Determination of PEG Ligands

The molecular weight of the polyethylene glycol (PEG) ligands grafted to the gold nanorods was determined by liquid chromatography–mass spectrometry (LC-MS) at the Systems Mass Spectrometry Core, Georgia Institute of Technology. PEG ligands were released from the gold nanorods by treatment with hydrogen peroxide and ammonium hydroxide prior to LC-MS analysis and the resulting extracts were analyzed by LC-MS. Data processing in Compound Discoverer yielded number- and weight-average molecular weights of *M*_n_ = 1.50 kDa and *M*_w_ = 1.63 kDa.

### CTAB-Functionalized AuNRs

Cetyltrimethylammonium bromide (CTAB)-functionalized gold nanorods were synthesized as previously described in the literature.^37^ Excess CTAB was removed by washing via centrifugation at 5,000 rpm for 5 min. The supernatant was carefully removed and the CTAB AuNRs were resuspended in the corresponding experimental solution. LPTEM imaging was performed using the protocol described above.

## Supplementary Material











## Data Availability

**Data and Code Availability**: The raw data sets generated during this study have been deposited on Hugging Face at https://huggingface.co/datasets/JamaliLab/Mod_NP_LPTEM/tree/main. The updated version of MoNet 2.0 used for this study can be found at https://github.com/JamaliLab/MoNet2.0.

## Notes

Ensemble averaging and convergence considerations for MSD-based metrics, statistics of e-tMSD and e-tMSAD diffusion parameters, ergodicity-breaking parameters, calculation of Debye length for each solution medium condition, zeta potentials: PEG-AuNRs and SiN_*x*_ membrane, angular displacement correction, correlating radial and angular displacements, theoretical stochastic diffusion processes and their simulations, collapsing e-tMSDs for FBM classified diffusion processes, statistical viscoelastic crossover parameters from translational and rotational analyses, fitting experimental viscoelastic moduli to a single-relaxation-time Maxwell model, comparing fitting of experimental viscoelastic moduli to Maxwell versus Jeffreys model, simulation of the mean squared displacements of a particle in a Maxwell fluid, effect of solution chemistry on diffusion of CTAB (cationic) Functionalized AuNRs (PDF)
Movie S1: 75 nm AuNR diffusing near the SiN_*x*_ membrane in H_2_O; Video playback is shown at 10× real-time speed (MP4)
Movie S2: 75 nm AuNR diffusing near the SiN_*x*_ membrane in 5 mM H_2_SO_4_; Video playback is shown at 10× real-time speed (MP4)
Movie S3: 75 nm AuNR diffusing near the SiN_*x*_ membrane in 1.5 mM NaCl; Video playback is shown at 10× real-time speed (MP4)
Movie S4: 75 nm AuNR diffusing near the SiN_*x*_ membrane in 5 mM PBS; Video playback is shown at 10× real-time speed (MP4)

## Notes

The preprint version of this work is available on ChemRxiv: Panicker, I.; Shabeeb, Z.; Hargus, C.; Jamali, V. Solution-Tunable Interfacial Interaction Landscape Governs Anomalous Nanoparticle Diffusion in Liquid-Phase Electron Microscopy. 2026; 1224. ChemRxiv. https://chemrxiv.org/doi/full/10.26434/chemrxiv-2025-60c5k/v3 (accessed April 16, 2026).

## References

[1] Shen H., Tauzin L. J., Baiyasi R., Wang W., Moringo N., Shuang B., Landes C. F. (2017). Single Particle Tracking: From Theory
to Biophysical Applications. Chem. Rev..

[2] Simon F., Weiss L. E., van Teeffelen S. (2024). A Guide to
Single-Particle Tracking. Nat. Rev. Methods
Primers.

[3] Jonge N. D., Ross F. M. (2011). Electron Microscopy
of Specimens in Liquid. Nat. Nanotechnol..

[4] Ross F. M. (2015). Opportunities
and Challenges in Liquid Cell Electron Microscopy. Science.

[5] Parent L. R., Bakalis E., Proetto M., Li Y., Park C., Zerbetto F., Gianneschi N. C. (2018). Tackling
the Challenges of Dynamic
Experiments Using Liquid-Cell Transmission Electron Microscopy. Acc. Chem. Res..

[6] Wu H., Friedrich H., Patterson J. P., Sommerdijk N. A., de Jonge N. (2020). Liquid-Phase Electron Microscopy for Soft Matter Science
and Biology. Adv. Mater..

[7] de
Jonge N., Houben L., Dunin-Borkowski R. E., Ross F. M. (2019). Resolution and Aberration Correction in Liquid Cell
Transmission Electron Microscopy. Nat. Rev.
Mater..

[8] Marolf D. M., Jones M. R. (2019). Measurement Challenges in Dynamic and Nonequilibrium
Nanoscale Systems. Anal. Chem..

[9] Moreno-Hernandez I. A., Crook M. F., Jamali V., Alivisatos A. P. (2022). Recent
Advances in the Study of Colloidal Nanocrystals Enabled by in Situ
Liquid-Phase Transmission Electron Microscopy. MRS Bull..

[10] Einstein A. (1905). Über
die von der molekularkinetischen Theorie der Wärme geforderte
Bewegung von in ruhenden Flüssigkeiten suspendierten Teilchen. Ann. Phys..

[11] Zheng H., Claridge S. A., Minor A. M., Alivisatos A. P., Dahmen U. (2009). Nanocrystal Diffusion in a Liquid Thin Film observed
by in Situ Transmission Electron Microscopy. Nano Lett..

[12] Ring E. A., de Jonge N. (2012). Video-Frequency Scanning
Transmission Electron Microscopy
of Moving Gold Nanoparticles in Liquid. Micron.

[13] Yuk J. M., Park J., Ercius P., Kim K., Hellebusch D. J., Crommie M. F., Lee J. Y., Zettl A., Alivisatos A. P. (2012). High-Resolution
EM of Colloidal Nanocrystal Growth Using Graphene Liquid Cells. Science.

[14] Chen Q., Smith J. M., Park J., Kim K., Ho D., Rasool H. I., Zettl A., Alivisatos A. P. (2013). 3D Motion
of DNA-Au Nanoconjugates in Graphene Liquid Cell Electron Microscopy. Nano Lett..

[15] Lu J., Aabdin Z., Loh N. D., Bhattacharya D., Mirsaidov U. (2014). Nanoparticle Dynamics in a Nanodroplet. Nano Lett..

[16] Woehl T. J., Prozorov T. (2015). The Mechanisms for Nanoparticle Surface Diffusion and
Chain Self-Assembly Determined from Real-Time Nanoscale Kinetics in
Liquid. J. Phys. Chem. C.

[17] Chen Q., Cho H., Manthiram K., Yoshida M., Ye X., Alivisatos A. P. (2015). Interaction
Potentials of Anisotropic Nanocrystals from the Trajectory Sampling
of Particle Motion using in Situ Liquid Phase Transmission Electron
Microscopy. ACS Cent. Sci..

[18] Verch A., Pfaff M., Jonge N. D. (2015). Exceptionally
Slow Movement of Gold
Nanoparticles at a Solid/Liquid Interface Investigated by Scanning
Transmission Electron Microscopy. Langmuir.

[19] Lin G., Chee S. W., Raj S., Král P., Mirsaidov U. (2016). Linker-Mediated Self-Assembly Dynamics
of Charged Nanoparticles. ACS Nano.

[20] Chee S. W., Baraissov Z., Loh N. D., Matsudaira P. T., Mirsaidov U. (2016). Desorption-Mediated
Motion of Nanoparticles at the
Liquid-Solid Interface. J. Phys. Chem. C.

[21] Fu X., Chen B., Tang J., Zewail A. H. (2017). Photoinduced Nanobubble-Driven
Superfast Diffusion of Nanoparticles Imaged by 4D Electron Microscopy. Sci. Adv..

[22] Cho H., Jones M. R., Nguyen S. C., Hauwiller M. R., Zettl A., Alivisatos A. P. (2017). The Use
of Graphene and Its Derivatives
for Liquid-Phase Transmission Electron Microscopy of Radiation-Sensitive
Specimens. Nano Lett..

[23] Tan S. F., Chee S. W., Lin G., Mirsaidov U. (2017). Direct Observation
of Interactions between Nanoparticles and Nanoparticle Self-Assembly
in Solution. Acc. Chem. Res..

[24] Lee J., Nakouzi E., Song M., Wang B., Chun J., Li D. (2018). Mechanistic Understanding
of the Growth Kinetics and Dynamics of
Nanoparticle Superlattices by Coupling Interparticle Forces from Real-Time
Measurements. ACS Nano.

[25] Cepeda-Pérez E., de Jonge N. (2019). Dynamics of Gold Nanoparticle
Clusters Observed with
Liquid-Phase Electron Microscopy. Micron.

[26] Chee S. W., Anand U., Bisht G., Tan S. F., Mirsaidov U. (2019). Direct Observations
of the Rotation and Translation of Anisotropic Nanoparticles Adsorbed
at a Liquid-Solid Interface. Nano Lett..

[27] Welling T. A., Sadighikia S., Watanabe K., Grau-Carbonell A., Bransen M., Nagao D., van Blaaderen A., van Huis M. A. (2020). Observation of Undamped 3D Brownian
Motion of Nanoparticles
Using Liquid-Cell Scanning Transmission Electron Microscopy. Part. Part. Syst. Charact..

[28] Yesibolati M. N., Mortensen K. I., Sun H., Brostrøm A., Tidemand-Lichtenberg S., Mølhave K. (2020). Unhindered Brownian Motion of Individual
Nanoparticles in Liquid-Phase Scanning Transmission Electron Microscopy. Nano Lett..

[29] Bakalis E., Parent L. R., Vratsanos M., Park C., Gianneschi N. C., Zerbetto F. (2020). Complex Nanoparticle
Diffusional Motion in Liquid-Cell
Transmission Electron Microscopy. J. Phys. Chem.
C.

[30] Cho H., Moreno-Hernandez I. A., Jamali V., Oh M. H., Alivisatos A. P. (2021). In Situ
Quantification of Interactions Between Charged Nanorods in a Predefined
Potential Energy Landscape. Nano Lett..

[31] Kang S., Kim J. H., Lee M., Yu J. W., Kim J., Kang D., Baek H., Bae Y., Kim B. H., Kang S. (2021). Real-Space Imaging of
Nanoparticle Transport and Interaction
Dynamics by Graphene Liquid Cell TEM. Sci. Adv..

[32] Jamali V., Hargus C., Ben-Moshe A., Aghazadeh A., Ha H. D., Mandadapu K. K., Alivisatos A. P., Designed A. P. A., Performed H. D. H. (2021). Anomalous
Nanoparticle Surface Diffusion
in LCTEM is Revealed by Deep Learning-Assisted Analysis. Proc. Natl. Acad. Sci. U. S. A..

[33] Xue C., Shi X., Tian Y., Zheng X., Hu G. (2020). Diffusion of Nanoparticles
with Activated Hopping in Crowded Polymer Solutions. Nano Lett..

[34] Ren A., Lu D., Wong E., Hauwiller M. R., Alivisatos A. P., Ren G. (2020). Real-time Observation of Dynamic
Structure of Liquid-Vapor Interface
at Nanometer Resolution in Electron Irradiated Sodium Chloride Crystals. Sci. Rep..

[35] Sarfati R., Schwartz D. K. (2020). Temporally Anticorrelated
Subdiffusion in Water Nanofilms
on Silica Suggests Near-Surface Viscoelasticity. ACS Nano.

[36] Liu S., Han X., Ophus C., Zhou S., Jiang Y.-H., Sun Y., Zhao T., Yang F., Gu M., Tan Y.-Z. (2023). Observing Ion Diffusion and Reciprocating Hopping Motion in Water. Sci. Adv..

[37] Shabeeb Z., Goyal N., Nantogmah P. A., Jamali V. (2025). Learning the Diffusion
of Nanoparticles in Liquid Phase TEM via Physics-Informed Generative
AI. Nat. Commun..

[38] Mandelbrotf B. B., Ness J. W. V. (1968). Fractional Brownian
Motions Fractional Noises and Applications. SIAM Rev..

[39] Goychuk I. (2009). Viscoelastic
Subdiffusion: From Anomalous to Normal. Phys.
Rev. E.

[40] Massignan P., Manzo C., Torreno-Pina J. A., García-Parajo M. F., Lewenstein M., Lapeyre G. J. (2014). Nonergodic Subdiffusion from Brownian
Motion in an Inhomogeneous Medium. Phys. Rev.
Lett..

[41] Pacheco-Pozo, A. ; Sokolov, I. M. ; Metzler, R. ; Krapf, D. Heterogeneous Diffusion in an Harmonic Potential: the Role of the Interpretation arXiv. 10.48550/arXiv.2505.13363

[42] Wang, A. ; Xu, M. ; Goel, R. ; Shabeeb, Z. ; Panicker, I. ; Jamali, V. SAM-EM Real-Time Segmentation for Automated Liquid Phase Transmission Electron Microscopy. arXiv, 2025. 10.48550/arXiv.2501.03153

[43] Metzler R., Jeon J. H., Cherstvy A. G., Barkai E. (2014). Anomalous Diffusion
Models and Their Properties: Non-stationarity, Non-ergodicity, and
Ageing at the Centenary of Single Particle Tracking. Phys. Chem. Chem. Phys..

[44] Efron B., Tibshirani R. (1986). Bootstrap Methods for Standard Errors, Confidence Intervals,
and Other Measures of Statistical Accuracy. Stat. Sci..

[45] Wang B., Kuo J., Bae S. C., Granick S. (2012). When Brownian Diffusion is not Gaussian. Nat. Mater..

[46] Scher H., Montroll E. N. (1975). Anomalous Transit-Time Dispersion in Amorphous Solids. Phys. Rev. B.

[47] Montroll E. W., Weiss G. H. (1965). Random Walks on
Lattices. Math
Phys..

[48] Muñoz-Gil G., Volpe G., Garcia-March M. A., Aghion E., Argun A., Hong C. B., Bland T., Bo S., Conejero J. A. (2021). Objective Comparison of Methods to Decode Anomalous Diffusion. Nat. Commun..

[49] Israelachvili, J. N. Intermolecular and Surface Forces; Academic press, 2011.

[50] Murashov V.
V., Leszczynski J. (1999). Adsorption
of the Phosphate Groups on Silica Hydroxyls:
An ab Initio Study. J. Phys. Chem. A.

[51] Edmond K. V., Elsesser M. T., Hunter G. L., Pine D. J., Weeks E. R. (2012). Decoupling
of Rotational and Translational Diffusion in Supercooled Colloidal
Fluids. Proc. Natl. Acad. Sci. U. S. A..

[52] Jawerth L., Fischer-Friedrich E., Saha S., Wang J., Franzmann T., Zhang X., Sachweh J., Ruer M., Ijavi M., Saha S., Mahamid J., Hyman A. A., Jülicher F. (2020). Protein Condensates
as Aging Maxwell Fluids. Science.

[53] Elbaum-Garfinkle S., Kim Y., Szczepaniak K., Chen C. C. H., Eckmann C. R., Myong S., Brangwynne C. P. (2015). The Disordered
P Granule Protein LAF-1 Drives Phase
Separation into Droplets with Tunable Viscosity and Dynamics. Proc. Natl. Acad. Sci. U. S. A..

[54] Mason T. G., Ganesan K., van Zanten J. H., Wirtz D., Kuo S. C. (1997). Particle
Tracking Microrheology of Complex Fluids. Phys.
Rev. Lett..

[55] Mason T. G. (2000). Estimating
the Viscoelastic Moduli of Complex Fluids Using the Generalized Stokes-Einstein
Equation. Rheol. Acta.

[56] Molaei M., Atefi E., Crocker J. C. (2018). Nanoscale
Rheology and Anisotropic
Diffusion Using Single Gold Nanorod Probes. Phys. Rev. Lett..

[57] Mason T. G., Weitz D. A. (1995). Optical Measurements of Frequency-Dependent Linear
Viscoelastic Moduli of Complex Fluids. Phys.
Rev. Lett..

[58] Cheng Z., Mason T. G. (2003). Rotational Diffusion Microrheology. Phys. Rev. Lett..

[59] Tirado M. M., Torre J. G. D. L. (1980). Rotational
Dynamics of Rigid, Symmetric Top Macromolecules.
Application to Circular Cylinders. J. Chem.
Phys..

[60] Tirado M. M., Martínez C. L., Torre J. G. D. L. (1984). Comparison of Theories for the Translational
and Rotational Diffusion Coefficients of Rod-like Macromolecules.
Application to Short DNA Fragments. J. Chem.
Phys..

[61] Rhodes F. H., Barbour C. B. (1923). The Viscosities
of Mixtures of Sulfuric Acid and Water. Ind.
Eng. Chem. Res..

[62] Sanfeliu-Cerdán N., Krieg M. (2025). The Mechanobiology of Biomolecular Condensates. Biophys. Rev..

[63] Maxwell J. C. (1867). On the Dynamical Theory of Gases. Philos. Trans. R. Soc..

[64] da
Silva N. R., Ferreira L. A., Mikheeva L. M., Teixeira J. A., Zaslavsky B. Y. (2014). Origin of Salt Additive Effect on Solute Partitioning
in Aqueous Polyethylene Glycol-8000–sodium Sulfate Two-phase
System. J. Chromatogr. A.

[65] Emilsson G., Schoch R. L., Feuz L., Höök F., Lim R. Y. H., Dahlin A. B. (2015). Strongly Stretched
Protein Resistant
Poly­(ethylene glycol) Brushes Prepared by Grafting-To. ACS Appl. Mater. Interfaces.

[66] Ferreira L. A., Teixeira J. (2011). Salt Effect on the
Aqueous Two-Phase System PEG 8000–Sodium
Sulfate. J. Chem. Eng. Data.

[67] Dumetz A., Lewus R. A., Lenhoff A. M., Kaler E. W. (2008). Effects of Ammonium
Sulfate and Sodium Chloride Concentration on PEG/Protein Liquid–Liquid
Phase Separation. Langmuir.

[68] Fritsch B., Körner A., Couasnon T., Blukis R., Taherkhani M., Benning L. G., Jank M. P., Spiecker E., Hutzler A. (2023). Tailoring
the Acidity of Liquid Media with Ionizing Radiation: Rethinking the
Acid-Base Correlation beyond pH. J. Phys. Chem.
Lett..

[69] Fritsch B., Lee S., Körner A., Schneider N. M., Ross F. M., Hutzler A. (2025). The Influence of Ionizing Radiation
on Quantification for In Situ and Operando Liquid-Phase Electron Microscopy. Adv. Mater..

[70] Salvo G. D., Merkens S., Körner A., Fritsch B., Malgaretti P., Hutzler A., Chuvilin A. (2025). A Workflow
for Modeling Radiolysis
in Chemically, Physically, and Geometrically Complex Scenarios. iScience.

[71] Parent L. R., Gnanasekaran K., Korpanty J., Gianneschi N. C. (2021). 100th Anniversary
of Macromolecular Science Viewpoint: Polymeric Materials by in Situ
Liquid-Phase Transmission Electron Microscopy. ACS Macro Lett..

[72] Dissanayake T. U., Wang M., Woehl T. J. (2021). Revealing Reactions
between the Electron
Beam and Nanoparticle Capping Ligands with Correlative Fluorescence
and Liquid-Phase Electron Microscopy. ACS Appl.
Mater. Interfaces.

