# Supplementary material for: Solution-Tunable Interfacial Interaction Landscape Governs Anomalous Nanoparticle Diffusion in Liquid-Phase Electron Microscopy
Source: ACS Nano. 2026 May 1;20(21):15550–64. doi: 10.1021/acsnano.6c04149 (PMC13235647; doi:10.1021/acsnano.6c04149)
Supplement: Supplementary file 1 [file nn6c04149_si_001.pdf]

Supporting Information: Solution-Tunable Interfacial Interaction Landscape Governs Anomalous Nanoparticle Diffusion in Liquid-Phase Electron Microscopy

Isabel Panicker,[†] Zain Shabeeb,[†] Cory Hargus,[‡] and Vida Jamali[*,†]

†*Georgia Institute of Technology, School of Chemical and Biomolecular Engineering, Atlanta, Georgia, 30332, United States of America*

‡*Entalpic, 5 Parv. Alan Turing, 75013 Paris, France*

E-mail: vida@gatech.edu

Ensemble Averaging and Convergence Considerations for MSD-Based Metrics

We note that ensemble-averaged MSD (eMSD) can be used to extract transport parameters such as the generalized diffusion coefficient and anomalous exponent. However, robustly extracting transport parameters from eMSD alone typically requires thousands of statistically independent trajectories to converge the underlying distributions, particularly in ergodicity-broken systems. Given the finite ensemble sizes and field of view imposed by LPTEM, such an analysis would not yield statistically converged or quantitatively reliable diffusion metrics in this context. Therefore, we restrict our extraction of important transport parameters to trajectory-resolved observables (*e.g.*, tMSD) which are better suited to the finite ensemble size of our experimental dataset.

Statistics of e-tMSD and e-tMSAD Diffusion Parameters

Table S1: Translational diffusion parameters with experimental values and 95% confidence intervals obtained from 2000 bootstrapping with replacement.

Condition	$\alpha_{\text{short time delay}}$, 95% CI	$\alpha_{\text{long time delay}}$, 95% CI
H_2O	0.45, [0.29, 0.56]	0.72, [0.59, 0.79]
5 mM H_2SO_4	0.53, [0.44, 0.63]	0.96, [0.77, 1.11]
1.5 mM NaCl	0.95, [0.83, 1.02]	N/A
5 mM PBS	0.98, [0.93, 1.03]	N/A

Table S2: Rotational diffusion parameters with experimental values and 95% confidence intervals obtained from 2000 bootstrapping with replacement.

Condition	$\alpha_{\text{short time delay}}$, 95% CI	$\alpha_{\text{long time delay}}$, 95% CI
H_2O	0.31, [0.18, 0.45]	0.55, [0.37, 0.69]
5 mM H_2SO_4	0.61, [0.46, 0.71]	0.94, [0.60, 1.11]
1.5 mM NaCl	0.78, [0.65, 0.86]	N/A
5 mM PBS	0.86, [0.80, 0.92]	N/A

Ergodicity-Breaking Parameters

The ergodicity-breaking parameters are calculated accordingly and shown in Figure S1:[1,2]
$\xi = \overline{\delta r^2(\tau)}/\langle\overline{\delta r^2(\tau)}\rangle$, $EB = \langle\xi^2\rangle - 1$.

To establish a baseline for interpreting these values, we computed EB parameters for 800 simulated trajectories of ergodic Brownian motion and weakly non-ergodic continuous-time random walk (CTRW) dynamics with an anomalous exponent, α, of 0.1. The simulated Brownian trajectories yielded an EB parameter of 0.29, providing an empirical upper bound for ergodic behavior. In contrast, the CTRW simulations resulted in an EB parameter of 1.30, serving as a reference for non-ergodic dynamics. Notably, the EB parameter observed for particles diffusing in 1.5mM NaCl and 5mM PBS is on par with the non-ergodic benchmark values, confirming the presence of weakly non-ergodic diffusion in these salt-containing environments. Additionally, particles diffusing in H_2O and 5mM H_2SO_4 have EB parameter values less than the empirical bound for ergodic behavior, further proving the distinction between modes of anomalous diffusion dependent on the ionic species available in solution.

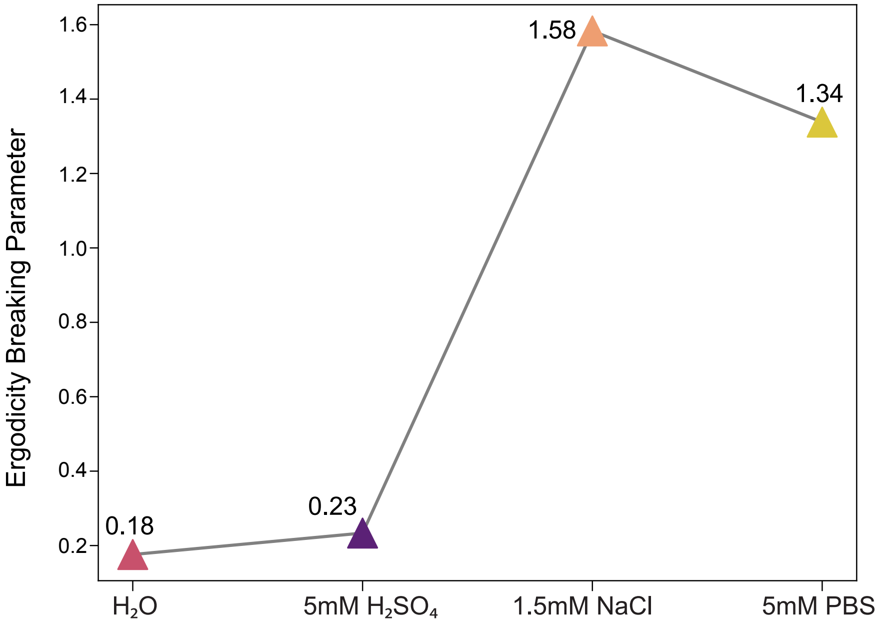

Figure S1: Ergodicity-Breaking Parameters of AuNR diffusion near the SiN_x membrane. Colors are indicative of diffusion media: H_2O (Orange), 5mM H_2SO_4 (Pink), 1.5mM NaCl (Purple), and 5mM PBS (Yellow).

Calculation of Debye Length for Each Solution Medium Condition

For aqueous solutions at a temperature of 25°C, the Debye length (κ^-1), can be simplified as:

$$\kappa^{-1} = \frac{0.304}{\sqrt{I}}, I = \frac{1}{2}\sum c_i z_i^2 \tag{S1}$$

where I is the ionic strength of the solution.[3,4]

In pure water, the Debye length is approximately 1000nm.[3]

For 5mM H_2SO_4, the first dissociation is strong and the second dissociation is defined by a dissociation constant, K_{a2}, with a value of 0.012 .[5] This results in final charge concentrations in solution of $H^+ = 0.008$M, ${HSO_4}^- = 0.002$M, and ${SO_4}^{-2} = 0.003$M. The ionic strength can then be calculated as

$$I = \frac{1}{2}(0.008(1^2) + 0.002(1^2) + 0.003(2^2)) \approx 1.1 \times 10^{-2}\ \text{M}. \tag{S2}$$

This results in a final approximate Debye length of 2.9nm.

In 1.5mM NaCl, total dissociation occurs, resulting in equal concentrations of positive and negative charges, therefore $I_{NaCl} = 1.5 \times 10^{-3}$ M and its Debye length (κ^{-1}) is approximately 7.9nm.

In 5mM PBS, the individual contributions from each ionic species after dilution are: Na^+ (4.58 mM), K^+ (0.23 mM), Cl^- (4.13 mM), $H_2PO_4^-$ (0.06 mM), and HPO_4^{2-} (0.25 mM). The

ionic strength is then calculated as

$$I = \frac{1}{2}\big(0.00458(1^2)+0.00023(1^2)+0.00413(1^2)+0.00006(1^2)+0.00025(2^2)\big) \approx 5.7\times10^{-3}. \quad \text{(S3)}$$

The Debye length of 5mM PBS is found to be approximately 4.0nm.

Table S3: Approximated Debye Lengths of Each Diffusion Medium

Condition	Debye Length, κ^{-1} (nm)
H_2O	1000
5mM H_2SO_4	2.9
1.5mM NaCl	7.9
5mM PBS	4.0

Zeta Potentials: PEG-AuNRs and SiN_x Membrane

We do not report a surface ζ value in nominally ion-free H_2O because the ionic strength is too low to support a stable electrokinetic determination, yielding non-reproducible measurements across replicates. To obtain a robust reference value under "water-like" conditions, we therefore measured the SiN_x surface in 0.5 mM KCl as a minimal supporting electrolyte needed to provide sufficient conductivity and a well-defined double layer for reproducible surface-ζ measurements in this method.

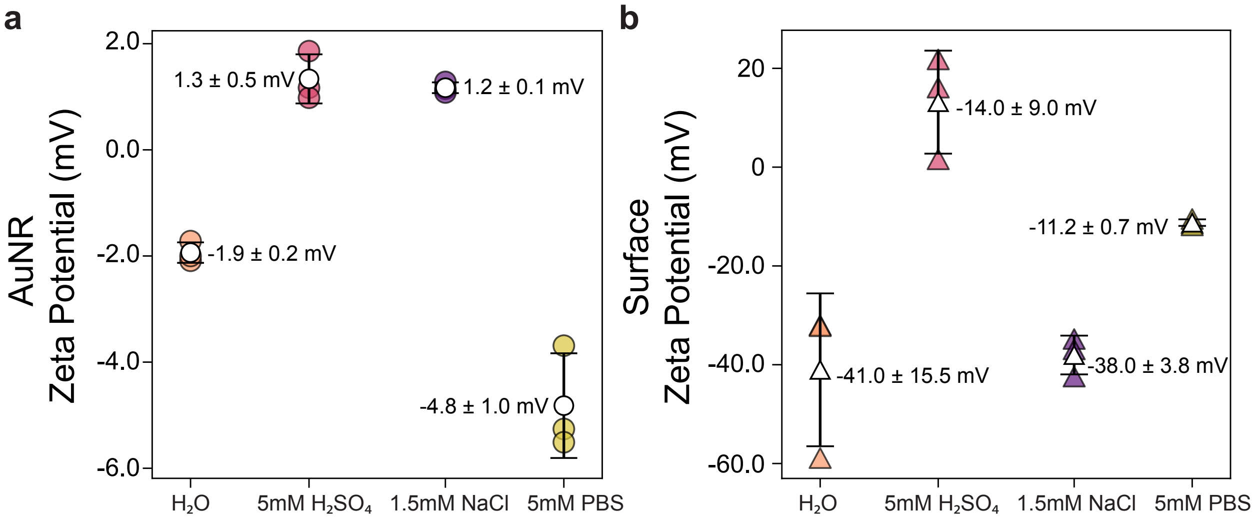

Figure S2: Zeta potentials of PEG-AuNRs and surface zeta potentials of silicon nitride membranes measured in respective diffusion media. Colors are indicative of diffusion medium: H_2O (Orange), 5mM H_2SO_4 (Pink), 1.5mM NaCl (Purple) and 5mM PBS (Yellow). Circular markers correspond to PEG-AuNRs and triangular markers correspond to silicon nitride membranes. Individual triplicate measurements are shown as colored markers, white markers represent the triplicate mean and whiskers indicate one standard deviation. (a) Zeta potentials of PEG-AuNRs in respective diffusion medium conditions. (b) Surface zeta potentials of silicon nitride in each respective diffusion medium conditions.

Angular Displacement Correction

To accurately calculate the time-lag dependent angular displacements used in the ensemble-averaged time-averaged mean squared angular displacement (e-tMSAD), angular trajectories were pre-processed to remove discontinuities caused by wrapping at $\pm 180°$.[6]

When measuring the orientation of anisotropic particles such as gold nanorods (AuNRs), frame-to-frame changes in angle θ can exhibit artificial discontinuities due to the bounded nature of angular measurements in the range $[-180°, 180°]$. To correct for this, we implemented a piecewise unwrapping scheme that adjusts each angular increment $\Delta\theta_t = \theta_{t+1} - \theta_t$ according to the following rule:

- If $-180° < \Delta\theta_t < -90°$, then $\Delta\theta_t^{\text{corrected}} = \Delta\theta_t + 180°$
- If $-90° \leq \Delta\theta_t \leq 90°$, then $\Delta\theta_t^{\text{corrected}} = \Delta\theta_t$
- If $90° < \Delta\theta_t < 180°$, then $\Delta\theta_t^{\text{corrected}} = \Delta\theta_t - 180°$
- If $\Delta\theta_t = \pm 180°$ or falls outside the above ranges, it is left unmodified.

This correction ensures that angular displacements are centered around $0°$, preserving physical continuity in rotational motion and avoiding jumps that can distort the calculated mean squared angular displacements. The corrected angular trajectory $\theta_t^{\text{corrected}}$ is then reconstructed by $\theta_{t+1}^{\text{corrected}} = \theta_t^{\text{corrected}} + \Delta\theta_t^{\text{corrected}}$, where $\theta_0^{\text{corrected}} = \theta_0$ (*i.e.*, the first frame angle remains unchanged). This method allows for accurate computation of angular displacements, enabling reliable analysis of rotational dynamics across all experimental conditions.

Correlating Radial and Angular Displacements

To see the effect of diffusing medium on the interactions between the AuNRs and SiN_x membrane and how it affects the particle's translational and rotational mobility, the respective translational and rotational displacements are correlated. Correlating translational and rotational displacements allows us to probe whether a particle's orientational dynamics are coupled to its lateral movement, which is an indicator of how interfacial constraints, such as pinning or confinement, govern overall nanoparticle mobility. A progressive increase in the Pearson correlation value, ρ, reflects the emergence of coordinated motion, while low or absent correlation is indicative of rotational pivoting or viscoelastic hindrance that decouples these degrees of freedom.[7–9]

The Pearson correlation, ρ, between the radial and angular frame-to-frame displacements is calculated for each of the 20 individual trajectories for the respective condition. Given the weakly non-ergodic nature of some diffusion processes and the variability across trajectories, ρ values are computed on a per-trajectory basis. This approach preserves trajectory-level information and avoids averaging across potentially distinct trajectories. The 20 resulting correlation coefficients of each trajectory per condition were then averaged, and the mean ρ values, along with their standard deviations, are shown in Figure S3.

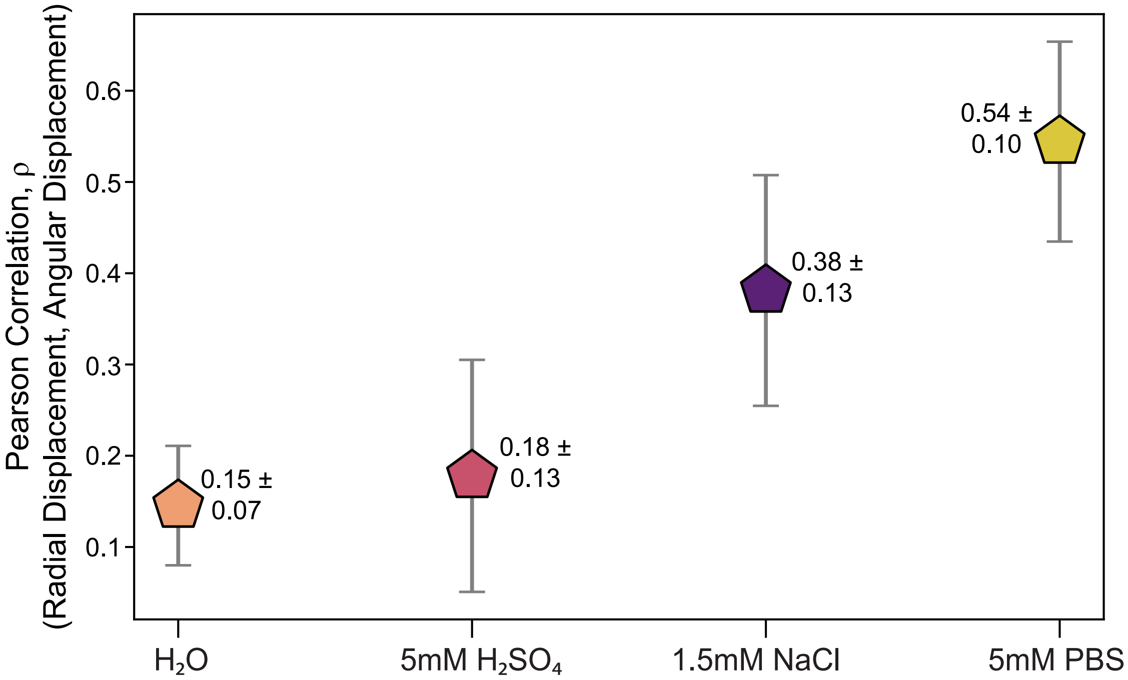

Figure S3: Averaged Pearson correlation values, ρ, between radial and angular displacements. Displacements were computed across 20 individual trajectories for each diffusion condition. For each trajectory the resulting ρ values were then averaged for each condition (H_2O, 5mM H_2SO_4, 1.5mM NaCl, and 5mM PBS). Error bars represent the standard deviation of ρ values across the 20 trajectories per condition. Colors are indicative of diffusion media: H_2O (Orange), 5mM H_2SO_4 (Pink), 1.5mM NaCl (Purple), and 5mM PBS (Yellow).

Theoretical Stochastic Diffusion Processes and Their Simulations

To train MoNet2.0, we generated a diverse dataset comprised of six stochastic diffusion processes: Brownian Motion (BM), Fractional Brownian Motion (FBM), Continuous Time Random Walk (CTRW), Annealed Transient Time Motion (ATTM), Scaled Brownian Motion (SBM), and Lévy Walk (LW), which are subsequently normalized by:

$$\mathbf{r}_{\text{normalized}} = \frac{\mathbf{r} - \min_t \mathbf{r}}{\max_{t,i} \mathbf{r} - \min_{t,i} \mathbf{r}} \tag{S4}$$

where the subscript t refers to the time frame, the subscript i refers to the axes, $\min_t \mathbf{r}$ denotes the per-axis minima, $\min_t \mathbf{r} = \begin{bmatrix} \min_t x_t & \min_t y_t \end{bmatrix}$, and $\max_{i,t} \mathbf{r}$ and $\min_{i,t} \mathbf{r}$ denote the maximum and minimum values across all entries in $\mathbf{r}$.

Below, we provide a brief theoretical description for each stochastic process and describe the methods used to simulate each process. For full theoretical details of simulating the anomalous diffusion processes (FBM, CTRW, ATTM, SBM, and LW), readers are referred to the Anomalous Diffusion (AnDi) Challenge and the corresponding article .[2]

Brownian Motion. Brownian motion describes a purely random process, where the particles undergo thermal motion.[10] The fundamental equation for Brownian Motion is:

$$\frac{\partial}{\partial t} P(x,t) = D \frac{\partial^2}{\partial x^2} P(x,t), \tag{S5}$$

where $P(x,t)$ is the probability density function for the particle that describes the position of a particle in position x at time t and D is the diffusion coefficient that is characteristic of the particle and its surrounding environment (particle geometry and surrounding temperature). Solving this equation with an initial condition of $x = 0$ at $t = 0$ (*i.e.*, $\Delta x_i = x_i$) with

unbounded x and t results in:

$$P(x,t) = \frac{1}{\sqrt{4\pi Dt}} \exp\left(-\frac{x^2}{4Dt}\right). \tag{S6}$$

The first moment (mean) of this distribution is zero, $\langle x(t) \rangle = 0$. The second moment of this distribution, *i.e.*, the variance $\langle x^2(t) \rangle$, equals the ensemble-averaged mean squared displacement (e-MSD) of the trajectories coming from this distribution and has the form:

$$\langle x^2(t) \rangle = \int_{-\infty}^{+\infty} x^2 \cdot \frac{1}{\sqrt{4\pi Dt}} \exp\left(-\frac{x^2}{4Dt}\right) dx = 2Dt. \tag{S7}$$

Trajectories were generated as discrete realizations of Brownian motion by summing Gaussian-distributed random displacements at each time step. For each trajectory, the displacements were sampled from a normal distribution with a zero mean and a variance proportional to the time step. The simulation iteratively updates the position as:

$$x_{i+1} = x_i + \mathcal{N}(0,1), \quad y_{i+1} = y_i + \mathcal{N}(0,1), \tag{S8}$$

where x_i and y_i are the current positions, and the displacements $\mathcal{N}(0,1)$ are sampled independently for x and y directions.

Fractional Brownian Motion. FBM generalizes Brownian motion by introducing memory effects in the step increments, characterized by the Hurst exponent $H = \frac{\alpha}{2}$.[1,11] The mean squared displacement (MSD) for particle position x scales as:

$$\langle x^2(t) \rangle \sim t^{\alpha}, \quad 0 < \alpha < 2, \tag{S9}$$

where $\alpha < 1$ corresponds to subdiffusion and $\alpha > 1$ to superdiffusion. FBM trajectories are generated using fractional Gaussian noise for both x and y directions. The process ensures

that the increments Δx and Δy are correlated in time according to H:

$$x_{i+1} = x_i + \eta_{x,i}(H), \quad y_{i+1} = y_i + \eta_{y,i}(H), \tag{S10}$$

where $\eta_{x,i}(H)$ and $\eta_{y,i}(H)$ are fractional Gaussian noise processes with memory effects determined by H. To train MoNet2.0, we sampled values of α (or equivalently $2H$) uniformly in the subdiffusive range of $[0.1, 1]$.

Continuous Time Random Walk. CTRW introduces waiting times between particle steps, where the waiting times $\psi(\tau_w)$ follow a power-law distribution:

$$\psi(\tau_w) \sim \tau_w^{-(1+\alpha)}, \quad 0 < \alpha < 1. \tag{S11}$$

This results in subdiffusive behavior, as particles remain trapped in localized regions for long periods before stepping.[1,12,13] To simulate CTRW trajectories, waiting times are drawn from the power-law distribution. After each waiting time, the particle position is updated with independent Gaussian random steps in both the x- and y-directions. The cumulative positions are then regularized to equally spaced time intervals. Specifically, the particle positions at time frame $i + 1$ are iteratively updated as:

$$x_{i+1} = x_i + \eta_{x,i}, \quad y_{i+1} = y_i + \eta_{y,i}, \tag{S12}$$

where $\eta_{x,i}$ and $\eta_{y,i}$ are independent Gaussian-distributed random steps applied after each waiting time. The resulting trajectories exhibit subdiffusion due to the broad distribution of waiting times. To train MoNet2.0, we sampled the values of α uniformly in the range of $[0.1, 1]$.

Lévy Walk. Lévy walks are stochastic processes that introduce long flights of constant

velocity.[14] The MSD scales as:

$$\langle x^2(t)\rangle \sim t^{\alpha}, \quad 1 < \alpha < 2. \tag{S13}$$

Here, α controls the distribution of step durations, leading to superdiffusive behavior when $1 < \alpha < 2$. To simulate Lévy walk trajectories, the step durations $\tau_{f,i}$ are drawn from a power-law distribution:

$$\phi(\tau_f) \sim \tau_f^{-(4-\alpha)}, \quad 1 < \alpha \leq 2, \tag{S14}$$

with the exponent defined by $\nu = 3 - \alpha$ (using a random ν when $\alpha = 2$). For each flight, a constant velocity, v, is sampled and a single flight direction, φ, is drawn uniformly from $\varphi = [0, 2\pi]$; the flight is discretized into an integer number of time steps, during which the particle positions are updated as

$$x_{i+1} = x_i + v\cos(\varphi), \quad y_{i+1} = y_i + v\sin(\varphi). \tag{S15}$$

To train MoNet2.0, values of α were sampled uniformly from $[1, 2]$.

Annealed Transient Time Model. ATTM describes a process in which the diffusion coefficient D fluctuates over discrete time intervals, leading to subdiffusive dynamics.[15,16] For a given D_i each time step is defined as

$$t_i = D_i^{-\gamma}, \tag{S16}$$

where $\gamma > 0$ is a scaling exponent that links the diffusion coefficient to the dwell time. Because t_i depends on the randomly sampled D_i, the time intervals vary across steps. The particle position is updated as

$$x_{i+1} = x_i + \sqrt{2D_i\, t_i}\,\eta_{x,i}, \quad y_{i+1} = y_i + \sqrt{2D_i\, t_i}\,\eta_{y,i}, \tag{S17}$$

where $\eta_{x,i}$ and $\eta_{y,i}$ are independent standard Gaussian random variables. To train MoNet2.0, values of α were sampled uniformly from $[0.1, 1]$.

Scaled Brownian Motion. SBM describes nonstationary diffusion in which the diffusion coefficient varies with time as[17]

$$D(t) \sim t^{\alpha-1}, \tag{S18}$$

so that the mean squared displacement scales as

$$\langle x^2(t) \rangle \sim t^{\alpha}. \tag{S19}$$

To simulate SBM, the variance is given by $\sigma^2 t^\alpha$; thus, if the variance at time step i is $\sigma^2 i^\alpha$, the position is updated as

$$x_{i+1} = x_i + \sqrt{\sigma^2 \Big[(i+1)^\alpha - i^\alpha\Big]}\,\eta_{x,i}, \quad y_{i+1} = y_i + \sqrt{\sigma^2 \Big[(i+1)^\alpha - i^\alpha\Big]}\,\eta_{y,i}, \tag{S20}$$

where $\eta_{x,i}$ and $\eta_{y,i}$ are independent standard Gaussian random variables. To train MoNet2.0, values of α were sampled uniformly from $[0.1, 1]$.

Collapsing e-tMSDs for FBM-Classified Diffusion Processes

To collapse the ensemble-averaged time-averaged MSD (e-tMSD) curves from FBM classified diffusion conditions onto a single master curve, each e-tMSD is first normalized by its own offset, δ_0^2, (the MSD at the first nonzero lag time) and its time axis by its characteristic time, τ_c. We chose the H_2O dataset as the reference curve and for the non-reference curve, τ_c is obtained by a one-parameter optimization in the log-log space that minimizes the root-mean-square distance to the reference curve.[18]

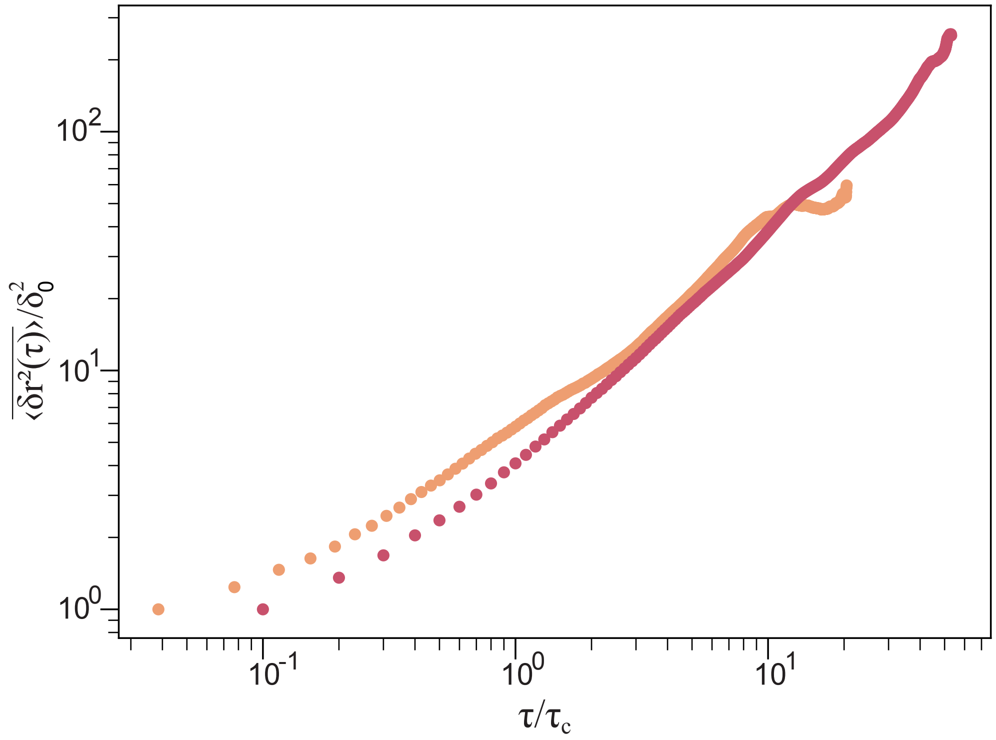

Figure S4: Collapsed e-tMSDs of FBM classified media using H_2O as the reference curve. Colors are indicative of diffusion media: H_2O (Orange) and 5mM H_2SO_4 (Pink).

Statistical Viscoelastic Crossover Parameters from Translational and Rotational Analyses

Table S4: Translational crossover relaxation times and moduli with 95% confidence intervals obtained from 2000 bootstrapping with replacement.

Condition	Relaxation Time, τ_c (s), 95% CI	Crossover Modulus (Pa), 95% CI
H_2O	0.37, [0.12, 2.34]	3068, [1711, 6726]
5 mM H_2SO_4	0.17, [0.10, 0.19]	5945, [5720, 11197]

Table S5: Rotational crossover relaxation times and moduli with 95% confidence intervals obtained from 2000 bootstrapping with replacement.

Condition	Relaxation Time, τ_c (s), 95% CI	Crossover Modulus (Pa), 95% CI
H_2O	0.87, [0.34, 2.82]	2884, [1642, 5046]
5 mM H_2SO_4	0.12, [0.10, 0.23]	5251, [3638, 7413]

Fitting Experimental Viscoelastic Moduli to a Single-Relaxation-Time Maxwell Model

To characterize the effective viscoelastic response within the two media that showcase FBM characteristics probed by nanoparticle motion, we model H_2O and 5mM H_2SO_4 frequency-dependent storage and loss moduli using a single-mode Maxwell model:[18–24]

$$G'(\omega) = \frac{(\tau_c\omega)^2 E}{1 + (\tau_c\omega)^2}, \qquad G''(\omega) = \frac{\tau_c\omega E}{1 + (\tau_c\omega)^2}, \tag{S21}$$

where $G'(\omega)$ and $G''(\omega)$ represent the storage and loss moduli, respectively; E is the high-frequency elastic modulus; and τ_c is the characteristic relaxation time ($\tau_c = 1/(2\pi f_c)$). To extract these parameters, we perform nonlinear least-squares fitting of the experimental moduli data to the above equations, using E and τ_c as free parameters.

Comparing Fitting of Experimental Viscoelastic Moduli to Maxwell vs Jeffreys Model

Two different rheological models were considered for the effective viscoelastic moduli of H_2O and H_2SO_4: single-relaxation-time Maxwell Fluid Model and Jeffreys Fluid Model. The single-relaxation-time Maxwell Fluid is modeled as mentioned in the previous section. The Jeffreys rheological model is given by:[18,25]

$$G'(\omega) = \frac{(\tau_c \omega)^2 E}{1 + (\tau_c \omega)^2}, \qquad G''(\omega) = \frac{\omega \tau_c E}{1 + (\omega \tau_v)^2} + \omega \lambda. \tag{S22}$$

where λ represents an additional viscosity term. For comparison, we evaluated the Jeffreys model by fixing $\lambda = 100$ Pa·s, a value large enough to produce a noticeable additional viscous contribution within our experimental frequency window. This allowed us to test whether including such a contribution improves the description of the data. The resulting model fits and residual values are shown in Figure S5. Quantitatively, the best fitting model to the effective viscoelastic moduli of H_2O and H_2SO_4 derived from both the e-tMSD and e-tMSAD is a single-relaxation-time Maxwell model.

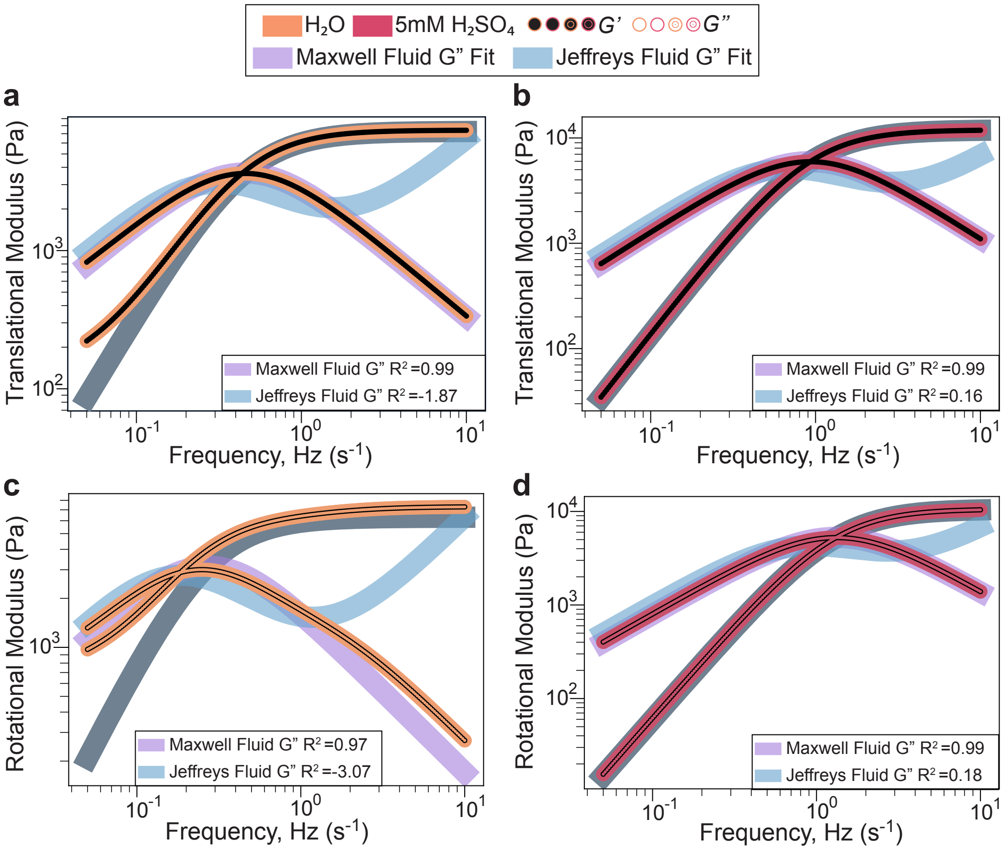

Figure S5: Effective viscoelastic Moduli of H_2O (Orange) and H_2SO_4 (Pink) derived from both e-tMSD and e-tMSAD, fit to a single-relaxation-time Maxwell Model (Light Purple) and a Jeffreys Model (Light Blue). Translational G' modulus is shown with black-filled markers, Translational G" modulus is shown with white-filled markers, Rotational G' modulus is represented by markers with a condition specific color scheme (orange for H_2O, pink for H_2SO_4) displayed in an orange-black-orange concentric pattern, Rotational G'' modulus is represented by markers with a condition specific color scheme (orange for H_2O, pink for H_2SO_4) displayed in an orange-white-orange concentric pattern. Resulting residual values of fits are shown.

Simulation of the Mean Squared Displacement of a Particle in a Maxwell Fluid

The mean-squared displacement (MSD) of a particle diffusing in a viscoelastic Maxwell fluid captures the time-dependent mechanical response of the surrounding environment. For a single-relaxation-time Maxwell fluid, the MSD can be approximated as:[18–20,24]

$$\langle \overline{\delta r^2(\tau)} \rangle = \delta_0^2 \left(1 + \frac{\tau}{\tau_c} \right), \tag{S23}$$

where δ_0^2 is the short-time diffusive amplitude and τ_c is the characteristic relaxation time of the medium. Nonlinear least-squares fitting is performed on the experimental ensemble time-averaged MSDs (e-tMSDs) to extract δ_0^2 and τ_c as fitting parameters.

Figure S6 shows representative fits of the experimental e-tMSDs to the approximation of a particle diffusing in a single-relaxation-time Maxwell fluid, along with residuals quantifying the deviation between model and data.

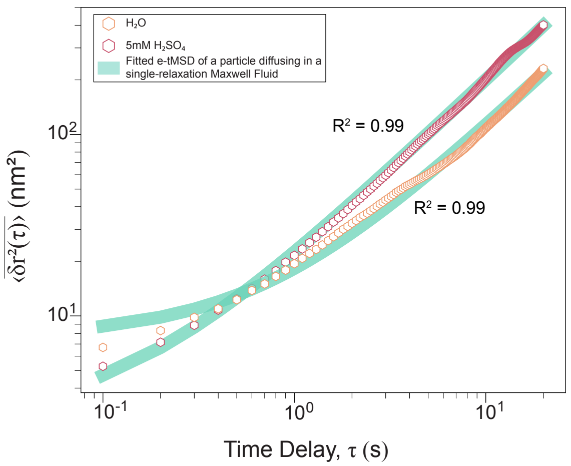

Figure S6: Experimental e-tMSDs of particle diffusing in respective medium conditions fit to the MSD of a particle diffusing in a viscoelastic Maxwell Fluid. Colors are indicative of diffusion media: H_2O (Orange) and 5mM H_2SO_4 (Pink). Overlaid lines in green represent a particle diffusing in a single-relaxation-time Maxwell fluid. Resulting residual values of fitting are also overlaid next to the respective diffusing medium conditions' MSD.

Effect of Solution Chemistry on Diffusion of CTAB (Cationic) Functionalized AuNRs

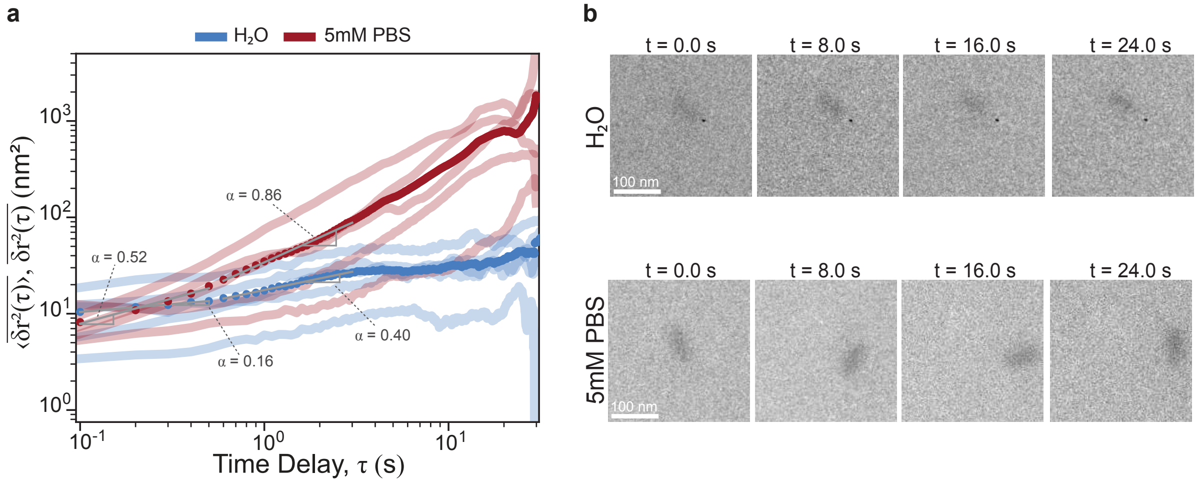

Figure S7: Statistical analysis of CTAB-Functionalized AuNR translational diffusion in each respective solution. Color indicates diffusion medium: H_2O (Blue), 5mM PBS (Red). (a) Log-log plot of time-averaged mean squared displacements (tMSDs) shown as transparent solid lines, grouped by medium, with the ensemble-average of tMSDs (e-tMSDs) overlaid as circular markers. Extracted anomalous exponents, α, and power-law fits (gray solid lines) for the short-time and long-time delay regimes are shown separately. (b) LPTEM image acquisitions of CTAB-coated gold nanorods diffusing in H_2O and 5mM PBS over time. All acquisitions were performed at 200kV, 100 fps (binned to 10 fps post-acquisition), and acquired at an electron flux of 30 $e^-/(\text{Å}^2 \cdot \text{s})$. Images shown above are cropped from 1024×1024 pixels (original acquisition image size) to 150×150 pixels. Scale bar is 100nm and is applied to all conditions.

Table S6: Anomalous Exponents and Generalized Diffusion Coefficients of CTAB Functionalized AuNRs in H_2O and 5mM PBS

Condition	$\alpha_{\text{short time delay}}$	$\alpha_{\text{long time delay}}$	D_α $[nm^2/s^\alpha]$
H_2O	0.16	0.40	3.8
5mM PBS	0.52	0.86	6.6

References

(1) Metzler, R.; Jeon, J. H.; Cherstvy, A. G.; Barkai, E. Anomalous diffusion models and their properties: Non-stationarity, non-ergodicity, and ageing at the centenary of single particle tracking. *Phys. Chem. Chem. Phys.* **2014**, *16*, 24128–24164.

(2) Muñoz-Gil, G. et al. Objective comparison of methods to decode anomalous diffusion. *Nat. Commun.* **2021**, *12*, 6253.

(3) Israelachvili, J. N. *Intermolecular and surface forces*; Academic press, 2011.

(4) Debye, P.; Hückel, E. Zur Theorie der Elektrolyte. *Physikalische Zeitschrift* **1923**, *24*, 185–206.

(5) CRC Handbook of Chemistry and Physics, 84th Edition Edited by David R. Lide (National Institute of Standards and Technology). CRC Press LLC: Boca Raton. 2003. 2616 pp. $139.95. ISBN 0-8493-0484-9. *Journal of the American Chemical Society* **2004**, *126*, 1586–1586.

(6) Mazaheri, M.; Ehrig, J.; Shkarin, A.; Zaburdaev, V.; Sandoghdar, V. Ultrahigh-Speed Imaging of Rotational Diffusion on a Lipid Bilayer. *Nano Lett.* **2020**, *20*, 7213–7219.

(7) Edmond, K. V.; Elsesser, M. T.; Hunter, G. L.; Pine, D. J.; Weeks, E. R. Decoupling of rotational and translational diffusion in supercooled colloidal fluids. *PNAS* **2012**, *109*, 17891–17896.

(8) Hargus, C.; Ghimenti, F.; Tailleur, J.; van Wijland, F. Odd dynamics of passive objects in a chiral active bath. *arXiv preprint arXiv:2412.20689* **2025**,

(9) Chee, S. W.; Anand, U.; Bisht, G.; Tan, S. F.; Mirsaidov, U. Direct Observations of the Rotation and Translation of Anisotropic Nanoparticles Adsorbed at a Liquid-Solid Interface. *Nano Lett.* **2019**, *19*, 2871–2878.

(10) Einstein, A. Über die von der molekularkinetischen Theorie der Wärme geforderte Bewegung von in ruhenden Flüssigkeiten suspendierten Teilchen. *Ann. Phys.* **1905**, *322*, 549–560.

(11) Mandelbrotf, B. B.; Ness, J. W. V. FRACTIONAL BROWNIAN MOTIONS, FRACTIONAL NOISES AND APPLICATIONS*. *SIAM Rev.* **1968**, *10*.

(12) Scher, H.; Montroll, E. N. Anomalous transit-time dispersion in amorphous solids. *Phys. Rev. B.* **1975**, *12*.

(13) Montroll, E. W.; Weiss, G. H. Random walks on lattices. II. . *Math. Phys.* **1965**, *6*, 167–181.

(14) Klafter, J.; Zumofen, G. Levy statistics in a Hamiltonian system. *Phys. Rev. E.* **1994**, *49*.

(15) Massignan, P.; Manzo, C.; Torreno-Pina, J. A.; García-Parajo, M. F.; Lewenstein, M.; Lapeyre, G. J. Nonergodic subdiffusion from brownian motion in an inhomogeneous medium. *Phys. Rev. Lett.* **2014**, *112*.

(16) Pacheco-Pozo, A.; Sokolov, I. M.; Metzler, R.; Krapf, D. Heterogeneous diffusion in an harmonic potential: the role of the interpretation. 2025; arXiv:2505.13363. arXiv. `http://arxiv.org/abs/2505.13363` (accessed April 14, 2026).

(17) Lim, S. C.; Muniandy, S. V. Self-similar Gaussian processes for modeling anomalous diffusion. *Phys. Rev. E* **2002**, *66*.

(18) Jawerth, L.; Fischer-Friedrich, E.; Saha, S.; Wang, J.; Franzmann, T.; Zhang, X.; Sachweh, J.; Ruer, M.; Ijavi, M.; Saha, S.; Mahamid, J.; Hyman, A. A.; Jülicher, F. Protein condensates as aging Maxwell fluids. *Science* **2020**, *370*, 1317–1323.

(19) Mason, T. G.; Ganesan, K.; van Zanten, J. H.; Wirtz, D.; Kuo, S. C. Particle Tracking Microrheology of Complex Fluids. *Phys. Rev. Lett.* **1997**, *79*, 3282–3285.

(20) Mason, T. G. Estimating the viscoelastic moduli of complex fluids using the generalized Stokes-Einstein equation. *Rheol. Acta* **2000**, *39*, 371–378.

(21) Elbaum-Garfinkle, S.; Kim, Y.; Szczepaniak, K.; Chen, C. C. H.; Eckmann, C. R.; Myong, S.; Brangwynne, C. P. The disordered P granule protein LAF-1 drives phase separation into droplets with tunable viscosity and dynamics. *PNAS* **2015**, *112*, 7189–7194.

(22) Molaei, M.; Atefi, E.; Crocker, J. C. Nanoscale Rheology and Anisotropic Diffusion Using Single Gold Nanorod Probes. *Phys. Rev. Lett.* **2018**, *120*.

(23) Sanfeliu-Cerdán, N.; Krieg, M. The mechanobiology of biomolecular condensates. *Biophys. Rev.* **2025**, *6*.

(24) Mason, T. G.; Weitz, D. A. Optical Measurements of Frequency-Dependent Linear Viscoelastic Moduli of Complex Fluids. *Phys. Rev. Lett.* **1995**, *74*, 1250–1253.

(25) Raikher, Y. L.; Rusakov, V. V.; Perzynski, R. Brownian motion in a viscoelastic medium modelled by a Jeffreys fluid. *Soft Matter* **2013**, *9*, 10857–10865.
